# Withaferin A inhibits Chikungunya virus nsP2 protease and shows antiviral activity in the cell culture and mouse model of virus infection

**DOI:** 10.1371/journal.ppat.1012816

**Published:** 2024-12-30

**Authors:** Kiran Bala Sharma, Chandru Subramani, Khashpatika Ganesh, Anshula Sharma, Brohmomoy Basu, Shivani Balyan, Ghanshyam Sharma, Shouri KA, Arundhati Deb, Mitul Srivastava, Saurabh Chugh, Sapna Sehrawat, Kanchan Bharadwaj, Archana Rout, Pankaj Kumar Sahoo, Suman Saurav, Rajender K. Motiani, Ramandeep Singh, Deepti Jain, Shailendra Asthana, Renu Wadhwa, Sudhanshu Vrati

**Affiliations:** 1 Regional Centre for Biotechnology, Faridabad, India; 2 Translational Health Science and Technology Institute, Faridabad, India; 3 National Institute of Advanced Industrial Science and Technology, Tsukuba, Japan; Duke University, UNITED STATES OF AMERICA

## Abstract

Chikungunya virus (CHIKV) is a mosquito-transmitted alphavirus causing fever, myalgia, and debilitating joint swelling and pain, which in many patients becomes chronic. The frequent epidemics of CHIKV across the world pose a significant public health burden necessitating the development of effective antiviral therapeutics. A cellular imaging-based high-content screening of natural compounds identified withaferin A (WFA), a steroidal lactone isolated from the plant *Withania somnifera*, as a potent antiviral against CHIKV. In the ERMS cells, WFA inhibited CHIKV replication early during the life cycle by binding the CHIKV non-structural protein nsP2 and inhibiting its protease activity. This inhibited the viral polyprotein processing and the minus-sense viral RNA synthesis. WFA mounted the nsP2 protease inhibitory activity through its oxidising property as the reducing agents N-acetylcysteine and Glutathione-monoethyl ester effectively reversed the WFA-mediated protease inhibition *in vitro* and abolished the WFA-mediated antiviral activity in cultured cells. WFA inhibited CHIKV replication in the C57BL/6 mouse model of chikungunya disease, resulting in significantly lower viremia. Importantly, CHIKV-infected mice showed significant joint swelling which was not seen in WFA-treated mice. These data demonstrate the potential of WFA as a novel CHIKV antiviral.

## Author summary

Chikungunya virus (CHIKV) spreads through the bites of the infected mosquitoes, and causes chikungunya fever involving severe joint and muscle pain. Epidemics of chikungunya fever are frequent, and the virus has become endemic in several regions. The virus has been reported from over 100 countries in Asia, Africa, South and Central America, and Europe, affecting almost half a million individuals annually. A CHIKV vaccine has recently been approved; however, no CHIKV-specific antiviral therapy is available. Considering the vast geographic spread of the virus and the large number of infections, developing novel CHIKV antivirals is an important public health priority. We screened small molecules from medicinal plants for anti-CHIKV activity and identified withaferin A (WFA) from the plant *Withania sominifera* as inhibiting virus replication in the cultured cells. CHIKV-infected mice treated with WFA showed reduced virus replication and no CHIKV infection symptoms. Our studies show that WFA binds the CHIKV protease and inhibits its action, which is necessary for producing the critical viral proteins for its replication. Our work has thus identified WFA as a novel CHIKV antiviral that may be studied further for its human use.

## Introduction

Chikungunya virus (CHIKV), a member of the Togaviridae family, is an arthropod-borne virus that primarily spreads through the bites of infected Aedes mosquitoes, notably *Aedes aegypti* and *Aedes albopictus* [1]. The virus is responsible for chikungunya fever, characterized by symptoms such as sudden onset of high fever, severe joint pain, muscle pain, headache, and rash. The fever and pain may persist for months, and fatalities have been reported occasionally [2]. The virus was first reported in Tanzania in 1952. A major outbreak of chikungunya fever occurred in India in 2006, where 1.25 million cases were reported [3]. In the last fifteen years, the virus has spread to over 110 countries in Asia, Europe, South and Central America, and Africa [4]. In 2023, ~460,000 chikungunya fever cases were reported worldwide [5]. Although CHIKV is not a deadly virus, it causes severe, long-lasting indisposition in a large population each year. The virus is listed by the Coalition for Epidemic Preparedness Innovations (CEPI) as a priority pathogen [6]. A live, attenuated CHIKV vaccine has recently been approved by the FDA [7,8]. However, no virus-specific therapy is available for the treatment of chikungunya fever. Greater attention is, therefore, required to develop novel antivirals against CHIKV.

CHIKV has a single-stranded, positive-sense RNA genome, which is ~12 kb long. During the virus replication, the genome encodes two polyproteins, namely, non-structural and structural polyproteins. The structural polyprotein is processed into five proteins (C, E3, E2, 6K, and E1) involved in the virion formation. The non-structural polyprotein is processed into four nonstructural proteins (nsP1, nsP2, nsP3, and nsP4) involved in different steps of virus replication. Among the known functions of alphavirus non-structural proteins, nsP1 is involved in mRNA capping, nsP2 has NTPase, helicase, and protease activity, nsP3 is the macrodomain, and nsP4 has the RNA-dependent RNA polymerase (RdRp) activity [2,9].

Several small drug-like molecule libraries, including the collection of the FDA-approved drug molecules, have been screened for anti-CHIKV activity using different methods [10–12]. Besides, computational methods have been used to identify and synthesize several designer drug molecules to inhibit CHIKV replication [13–15]. Like many other viruses, CHIKV infection and replication involve a variety of viral and host proteins. Accordingly, all steps of the virus life cycle, including most of the viral and several host proteins involved in these steps, have been targeted. Thus, compounds targeting the viral structural proteins E2, Capsid, and 6K, and non-structural proteins nsP1, nsP2, and nsP4 were shown to have anti-CHIKV activity. Similarly, several compounds targeting the host processes, such as membrane fusion, lipid pathways, pyrimidine and purine synthesis pathways, protein synthesis, cellular proteins and enzymes, showed anti-CHIKV activity. Several review articles listing these molecules have been published in recent years [10–12,15–21]. The anti-CHIKV activity of these molecules was demonstrated in the cell culture infection model of CHIKV, and only a handful of these were tested in an animal model.

We have screened a collection of drug-like compounds obtained from natural sources and have identified Withaferin A (WFA) as a potent inhibitor of CHIKV replication in different cell types. The antiviral activity of WFA was demonstrated in the mouse model of CHIKV infection. The antiviral action of WFA was related to its ability to bind the nsP2 protein and inhibit its protease activity.

## Material and methods

### Ethics statement

For the animal experiments, the guidelines on the care and use of laboratory animals provided by the Committee for the Purpose of Control and Supervision of Experiments on Animals (CPCSEA), Government of India, were followed. The experimental protocol was approved by the Institutional Animal Ethics Committee of Regional Centre for Biotechnology (RCB/IAEC/2019/047).

### Cells and viruses

The Vero, BHK-21, Huh7, and HeLa cell lines were obtained from the cell repository at the National Centre for Cell Sciences, Pune, India. The human embryonal rhabdomyosarcoma (ERMS) (RD-CCL-136-ATCC) and mouse myoblast C2C12 cells (C2C12-CRL-1722-ATCC) were obtained from the ATCC, USA. Dulbecco’s modified Eagle medium (DMEM) (HiMedia; AL007A) was used to culture ERMS, C2C12, Huh7, and HeLa cells. Minimum essential medium Eagle (MEM) (HiMedia; AL0475) was used for culturing Vero and BHK-21 cells. All the media were supplemented with 10% fetal bovine serum (FBS) (Gibco; 10270106) and 1x penicillin-streptomycin-glutamine (PS) (HiMedia; A001A). The cells were grown at 37°C under a 5% CO_2_ atmosphere.

The CHIKV-LR-5’GFP virus was generated from the synthetic plasmid DNA (Epoch Life Science, Inc., USA) as described before [22]. The recombinant virus was based on the LR2006 OPY1 strain of CHIKV [22]. The CHIKV-LR-5’GFP was used for the screening assay. Whereas the CHIKV isolate IND-06-Guj was used for the validation and all subsequent studies including the animal experiments. The BHK-21 cell line was used for culturing CHIKV, and Vero cells were used for the plaque assays to determine the virus titer.

### Reporter virus generation

The double subgenomic infectious clone of the LR2006 OPY1 strain of CHIKV [22] was used to construct the recombinant CHIKV-LR 5’ Green fluorescent protein (GFP) virus. In CHIKV-LR 5’GFP, the GFP is located 5’ to the cDNA encoding the viral structural proteins under a copy of the subgenomic promoter. The synthetic plasmid representing the virus infectious clone was *in vitro* transcribed from the SP6 promoter using the mMESSAGE mMACHIN SP6 Transcription Kit (Invitrogen; AM1340). BHK-21 cells (6x10^5^ cells) were transfected with 10 μg RNA using Lipofectamin 2000 (Invitrogen; 11668027) as per the manufacturer’s protocol. At 48 and 72 h post-transfection, the culture supernatant was harvested and titrated by plaque assay. The GFP expression in the infected cells was monitored using a Nikon Eclipse Ts2 fluorescence microscope. Subsequently, a 500 μl culture supernatant was used to infect a 75- cm^2^ tissue culture flask of BHK-21 cells in a 15 ml medium. The culture supernatant was collected at 36 h post-infection (pi) and stored in small aliquots at -70°C. The virus titre was determined by plaque assay and expressed as plaque-forming units (PFU)/ml.

### Collection of the natural compounds

MTT-based cell viability assay was performed on 85 compounds derived from herbs used in traditional home medicine systems, and 66 compounds (S1 Table) were selected that were not toxic at 1 μM concentration using human breast cancer (MCF7) and bone cancer (U2OS) cells, both possessing wild type p53 similar to the normal cells. All compounds used in the screening were procured in their purified form (95% purity) from commercial suppliers such as Sigma, Wako and Tokiwa phytochemicals, Japan. WFA used in the experiments was >95% pure and procured from Sigma (Cat no. 89910).

### Cell-based antiviral screening assay

BHK-21 and ERMS cells were used for the screening assay for which various conditions, such as the cell density, viral-infective dose as the multiplicity of infection (MOI) and assay end-point, were standardised. Ribavirin was used as the positive control since it has been shown to inhibit CHIKV *in vitro* and *in vivo* [23–25]. The validation of the assay was done by studying the quality parameters such as signal-to-noise (S/N) ratio, coefficient of variation (CV %) and Z’ factor to ensure a reliable conclusion [26].

The cells were seeded overnight onto a 96-well clear bottom black plate (Corning) at a density of 10,000 cells/well in the culture media supplemented with 2% FBS. The test compounds were dissolved in DMSO. The cell monolayers were treated with the test compounds at the final concentration of 10 μM. The control cells were treated similarly with DMSO. Immediately after adding the compounds, BHK-21 or ERMS cells were infected with CHIKV-LR 5’ GFP at 0.1 or 5 MOI and incubated for 20 or 32 h, respectively, at 37°C under 5% CO_2_ atmosphere. At the respective endpoints, the cell nuclei were stained by incubating the cells with 1 μg/ml Hoechst stain for 15 min at 37°C and 5% CO_2_. Following the nuclei staining, the images were acquired with channels for Hoechst and GFP using the ImageXpress High-Content Imaging system (Molecular Devices) (S1 Fig). We acquired 16 images/well covering ~95% of the well using a 10x-magnifying objective and analysed them with multi-wavelength cell scoring analysis software (MetaXpress software; Molecular Devices). The cell viability was calculated from the nuclei counts in the test substance- and DMSO-treated cells and the inhibition of the virus replication was calculated based on the GFP fluorescence intensity in the test substance- and DMSO-treated cells. The primary screen identified compounds showing >80% cell viability and >80% virus inhibition at 10 μM concentration. We performed a secondary screen with these compounds at lower concentrations (1 μM, 0.5 μM, and 0.1 μM) in the same cell lines.

### *Animal* experiment

The IND-06-Guj strain of CHIKV was used for the animal experiments. The C57BL/6 mice of either sex, 8–10 weeks old with 18–22 g weight, were divided into four groups of 10 animals each. Group 1 (Mock) mice were mock-infected with PBS; Group 2 (Mock+WFA) mice were mock-infected with PBS and treated with WFA; Group 3 (CHIKV+Vehicle) mice were infected with CHIKV and treated with the vehicle, and Group 4 (CHIKV+WFA) mice were infected with CHIKV and treated with WFA. The mice were infected sub-cutaneous with 10^4^ PFU of CHIKV (IND-06-Guj strain) in 50 μl PBS on the ventral side of both hind limbs towards the ankle. The mice were treated by the intraperitoneal (I.P.) route twice daily with the vehicle (PBS+0.5% CMC+26% Ethanol) or WFA dissolved in the vehicle. The first dose of WFA was delivered 4 h pi. The mice were followed for two weeks, during which the body weight and paw edema were measured daily using a digital plethysmometer. The blood was collected each day for the first 4 days and stored as the serum to measure viremia.

### Virus attachment and entry assays

To study the effect of WFA on CHIKV attachment to the host cells, the ERMS cell monolayer was treated with 1 μM WFA for 2 h at 37°C and then incubated with CHIKV (1 MOI) on ice for 1 h to promote the virus attachment without entering the host cell. The cells were then washed with ice-cold PBS and lysed with RNAiso Plus (Takara; 9109) for RNA isolation. To study the effect of WFA on CHIKV entry, the ERMS cell monolayer was incubated with 1 μM WFA for 2 h. The cell monolayer was then incubated with CHIKV (1 MOI) for 1 h on ice. This was followed by the cell monolayer being washed with ice-cold PBS and incubation at 37°C for 1 or 2 h. The cells were treated with trypsin (2.5 g/L) for 1 min and washed with PBS to remove the uninternalized virion particles. The cells were lysed using RNAiso Plus (Takara; 9109) for RNA isolation. The viral RNA levels were determined by qRT-PCR.

### CHIKV RNA transfection and virus infection assessment

CHIKV RNA was isolated from the purified virions using the RNeasy kit (Qiagen; 74104). ERMS cells were transfected with 200 ng viral RNA using Lipofectamine 2000 (Invitrogen; 11668027). To confirm the viral RNA transfection and establishment of infection, cells were harvested at 6 h post-transfection (pt), and viral RNA levels were determined by qRT-PCR. To study the role of WFA in CHIKV replication, the viral RNA-transfected ERMS cells were treated with WFA (1 μM) or DMSO (control) at 6 h pt and harvested at 24 h pt. The cells were lysed in RNAiso Plus (Takara; 9109) for RNA isolation, and the levels of CHIKV RNA were determined by qRT-PCR. The culture supernatants were collected to measure the viral titer by plaque assay.

### RNA isolation and qRT-PCR

The total RNA from the cells was extracted by lysis in the RNAiso Plus (Takara; 9109) reagent, and 500 ng RNA was used for cDNA preparation using random hexamers and ImProm-II reverse transcription system (Promega; A3800). The qPCR reactions were performed using the 2x SYBR-green reagent (Takara; RR420A) in the QuantStudio 6 flex RT-PCR machine. The *Gapdh* levels were used as the internal housekeeping control. The PCR conditions were as follows: 94°C for 2 min (1 cycle), 94°C for 15 sec, 55°C for 30 sec, 72°C for 1 min (40 cycles). All experiments had biological duplicates and were performed independently three or more times, and the qPCR for each sample was done in technical triplicates. The fold-change in the RNA level is represented as the mean ± SD of three or more independent experiments. The primers (5’-3’) used in the study were as follows: GAPDH: F-TGCACCACCAACTGCTTAGC; R-GGCATGGACTGTGGTCATGAG; CHIKV: F- GGCAGTGGTCCCAGATAATTCAAG; R- GCTGTCTAGATCCACCCCATACATG; SINV: F- AAAGGATACTTTCTCCTCGC; R- TGGGCAACAGGGACCATGCA; JEV: F-AGAGCACCAAGGGAATGAAATAGT; R- AATAAGTTGTAGTTGGGCACTCTG; DENV-2: F- CAATATGCTGAAACGCGAGAGAAA; R-CCCCATCTATTCAGAATCCCTGCT.

### Strand-specific quantitation of CHIKV RNA

The plus- and minus-sense RNA of CHIKV was quantified using a strand-specific qRT-PCR assay as described previously [27]. The total RNA isolated from the CHIKV-infected cells was employed for the cDNA synthesis by reverse transcription. Tagged (non-viral sequence) primers PtagCHIKp and NtagCHIKn were used for the cDNA synthesis from the plus- and minus-sense CHIKV RNA, respectively. The real-time qPCR was performed using a combination of primers that bind to the non-viral tag sequence and viral strand as shown in S2 Table. The strand-specific RNA copy numbers were determined using the plus- and minus-sense RNA-specific standard curves. For making the standard curve, the total RNA isolated from the CHIKV-infected cells was used for the cDNA synthesis using the random hexamers. The PCR was done with the T7-tagged primers (S2 Table). The CHIKV nsP2 RNA was generated by *in vitro* transcription of the above PCR products. The RNA was quantified using a spectrophotometer and subjected to qPCR. The standard curve was plotted using the Ct values obtained from the range of known RNA concentrations and the calculated copy number of strand-specific CHIKV RNA.

### Computational analysis

The CHIKV nsP2 protein structure (PDB-ID 3TRK, resolution 2.40 Å) was retrieved from the RCSB protein data bank. We used the amino acid numbering based on the CHIKV nsP2 protein sequence rather than the numbering used in the crystal structure based on the polyprotein. The structure of ligands WFA and Withanone (Wn) were retrieved from the PubChem database. The molecular docking was conducted to study the WFA or Wn binding to CHIKV nsP2 using the Glide module in Schrodinger [28]. Clustering of the docked poses was conducted to generate the most likely complex of WFA-nsP2 based on several conformers and the lowest binding energy. SiteMap [29] was then used to cross-check the findings of the molecular docking. Both the methods identify the potential ligand interacting sites on the protein’s surface. The molecular dynamics (MD) simulations were carried out to assess the stability of the ligands at the potential binding sites on nsP2 using the AMBER22 package. The solute was placed within a cubic box, ensuring a minimum distance of 12.0 Å between any protein atom and the edge of the box filled with explicit water molecules (TIP3P), and counter-ions were added. The rest of the parameters were the default for any standard protein-ligand simulation.

### Expression and purification of CHIKV nsP2 protein

The CHIKV nsP2 cDNA was codon-optimized and synthesised commercially (GenScript). The cDNA was cloned in the pET28a vector using the gene-specific primers. From here, the CHIKV nsP2 cDNA, or the cDNA encoding its protease and helicase domains were sub-cloned into a modified pET14b vector containing the N-terminus His_6_-SUMO tag followed by the PreScission protease site. The desired amino acid residue in the CHIKV nsP2 cDNA, cloned in the expression plasmid, was mutated by the site-directed mutagenesis method described below. The expression plasmid was transferred to *Escherichia coli* Rosetta (DE3) cells. The transformed bacteria were grown in the Luria-Bertani (LB) medium till OD600 reached 0.6–0.8, followed by the protein induction using 0.5 mM isopropyl β-d-1-thiogalactopyranoside (IPTG) at 18°C for 16 h. The cells were harvested by centrifugation and resuspended in the lysis buffer (20 mM HEPES pH 7.5, 500 mM NaCl, 5% Glycerol and 2 mM β-mercaptoethanol) for sonication, and the lysate was centrifuged at 15,000 rpm for 1 h. The supernatant was filtered and loaded onto the high trap Ni-NTA column (Cytiva). The protein was eluted using a gradient of imidazole. The PreScission protease was added to the eluted proteins to cleave the His6-SUMO tag and dialysed overnight at 4°C. The tag-cleaved protein was concentrated and subjected to the size-exclusion chromatography using the Superdex 200 16/600 (Cytiva) column equilibrated with a buffer containing 20 mM Tris pH 7.5, 300 mM NaCl, 5% glycerol, and 0.5 mM DTT. The eluted protein was concentrated, run on an SDS-PAGE to check the quality (S2 Fig), and stored at -80°C. The purified proteins were Western blotted with anti-His antibody (Thermo Fisher, MA1-21315) to rule out the contamination with the His_6_-SUMO tagged protein.

### Site-directed mutagenesis

The site-directed mutagenesis was done by the polymerase chain reaction (PCR) using the overlapping primers on the expression plasmid containing the CHIKV nsP2 cDNA (see above). The overlapping primers were designed according to the desired nucleotide changes in the coding region of the protein. The PCR was carried out using Phusion polymerase (NEB, M0530S). The PCR product was treated with DpnI (NEB, R0176S) to digest the methylated parent plasmid (the template). The resultant product was desalted and purified by a PCR purification kit (Qiagen, 28506). The resultant DNA was used to transform the E. coli DH5α competent cells that were grown at 37°C. The plasmid was isolated and subjected to nucleotide sequencing to verify the mutation.

### Microscale thermophoresis (MST) experiments

The interaction of WFA and Wn with the CHIKV nsP2 or its protease and helicase domains was studied using the MST assay. For the MST experiments, 10 μM protein was labelled with MO-L011-Red NHS fluorescent dye using the Monolith NT protein labelling kit (NanoTemper Technologies). The labelled protein (1 μM) was incubated with various concentrations of WFA and loaded into the Monolith NT.115 capillaries. The data was collected using the Monolith NT.115 device with a 60% (RED) LED power and analysed using MO affinity analysis v2.3 software. The difference in fluorescence (ΔF) was calculated by measuring the fluorescence of protein in only buffer and the fluorescence of protein with WFA and plotted against WFA concentration. For the binding affinity analysis, the ligand-dependent changes in the temperature-related intensity change (TRIC) https://nanotempertech.com/nanopedia/tric/are plotted as F_norm_ values *vs*. ligand concentration in a dose-response curve. The F_norm_ values are plotted as parts per thousand (‰). For each trace, the F_norm_ value for the dose-response curve is calculated by dividing F_1_ by F_0_. F_1_ corresponds to the fluorescence value measured in the heated state, while F_0_ is measured in the cold state before the IR laser is turned on [30].

### FRET-based CHIKV nsP2 protease assay

The proteolytic activity of the CHIKV nsP2 protease was determined using a FRET-based approach [31,32]. The assay was performed *in vitro* using the purified CHIKV nsP2 proteins and the FRET-based octapeptide substrates- {DABCYL}-RAGA↓GIIET-{Glu(EDANS)}-NH2 (nsp1/2 site), {DABCYL}-RAGC↓APSYR-{Glu(EDANS)}-NH2 (nsp2/3 site), and {DABCYL}-RAGG↓YIFSS-{Glu(EDANS)}-NH2 (nsp3/4 site) (Biolink) [33]. A random sequence containing peptide {DABCYL}-GYFAGSRIS-{Glu(EDANS)}-NH2 was used as a negative control to demonstrate the enzyme specificity for the peptide substrate.

To study the protease activity, 1 μM CHIKV nsP2 was added to a Nunc 96-well black plate with different concentrations of the peptide substrate in the assay buffer (20 mM Bis-Tris-Propane, pH 8). The protease activity was monitored by reading the fluorescence at regular intervals using a multimode plate reader SpectraMax i3x at an excitation wavelength of 340 nM and emission wavelength of 490 nM. For the protease inhibition assay, 1 μM CHIKV nsP2 was incubated with different concentrations of WFA in the assay buffer for 30 min at 25°C. Following the incubation, the protein-WFA mix was added to a Nunc 96-well black plate with 25 μM peptide substrate in the assay buffer (20 mM Bis-Tris-Propane, pH 8) and the fluorescence was measured at regular intervals. A substrate control reaction measured the auto-fluorescence generated by just the substrate. Wn was used at 10 μM concentration as a negative control for the inhibition assay. The CHIKV nsP2-helicase domain (1 μM) was used as a negative control for the protease assay.

To evaluate the effect of NAC on the protease activity, 1 μM CHIKV nsP2 was incubated with 5 μM WFA and 1- or 2-mM NAC in the assay buffer for 30 min at 25°C. The assay fluorescence was measured at regular intervals.—All enzyme reactions were performed in duplicates and two independent experiments. The average fluorescence of each sample was calculated and plotted in the graph.

### Statistical analysis

The statistical analysis was done using the paired Student’s t-test or one-way ANOVA followed by Tukey’s post-hoc test. The data significance was considered at *p-*values of <0.05.

## Results

### Development of an imaging-based CHIKV antiviral screening assay

To discover novel antivirals against CHIKV, we utilised an imaging-based virus infection assay of the cell using a green fluorescent protein (GFP)-expressing reporter virus, CHIKV-GFP. The expression of GFP following the virus infection was monitored employing an imaging platform and used to quantify the degree of CHIKV infection in the cells. The nuclei staining determined the cell viability, as described in the methods. We standardised the assay conditions, such as the cell density, MOI, and assay end-point in different cell lines. Finally, the assay was performed in a 96-well plate with 10,000 cells/well, infected at 0.1 or 5 MOI for 20 or 32 h in BHK-21 or ERMS cells, respectively. The signal-to-noise (S/N) ratio for the assay was between 40–60, the coefficient of variation (CV) was <1.9% and the Z’ factor was 0.91, confirming the robustness, sensitivity, accuracy, and reproducibility of the assay for the large-scale antiviral screening.

### Screening of a collection of natural compounds for the anti-CHIKV activity

**Fig 1:**
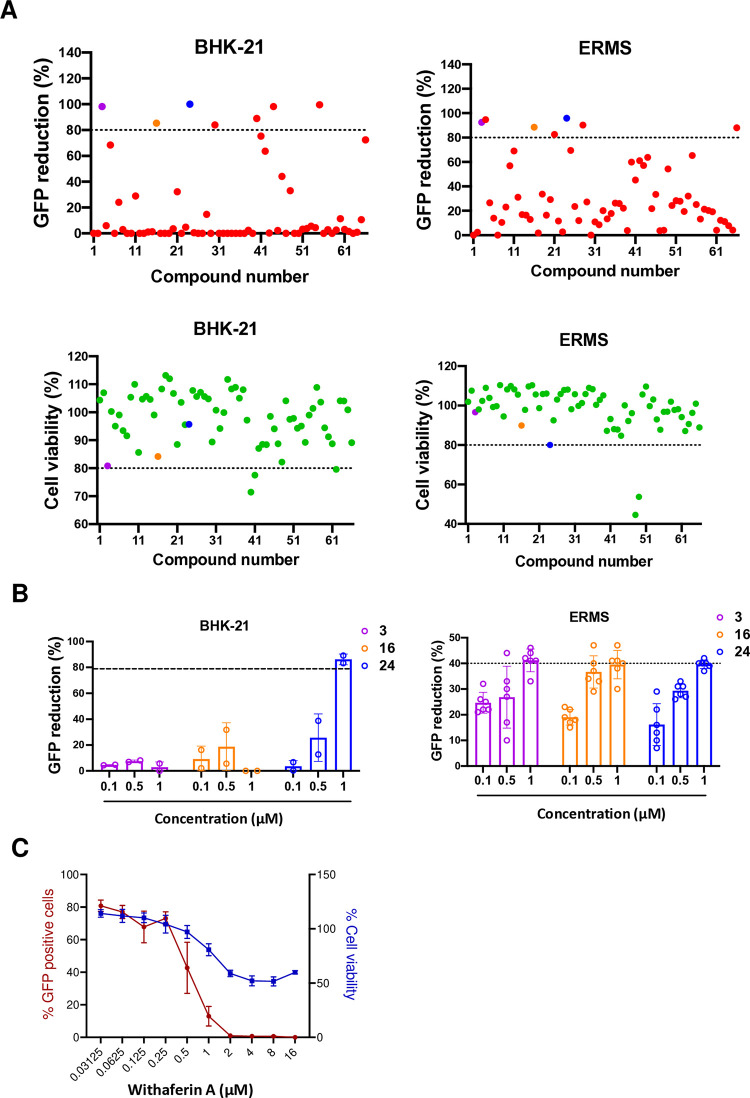
Screening of the natural compounds for the CHIKV antiviral activity. (A) BHK-21 or ERMS cells were seeded in a 96-well plate and infected with CHIKV-GFP at 0.1 or 5 MOI for 20 or 32 h, respectively and treated with DMSO (vehicle control) or the test compounds. The top panels show the CHIKV replication inhibition as per cent GFP reduction using different compounds at 10 μM concentration. The bottom panels show the percentage of cell viability for each compound. (B) BHK-21 or EMRS cells were seeded in a 96-well plate and infected with CHIKV-GFP at 0.1 or 5 MOI for 20 or 32 h, respectively and treated with the indicated concentrations of different compounds. The bar graphs show the CHIKV replication inhibition as per cent GFP reduction for the selected compounds at lower concentrations of 0.1, 0.5, and 1.0 μM. (C) BHK-21 cells were infected with CHIKV-GFP (0.1 MOI) and treated with different concentrations of WFA. The dose-response curve demonstrating the per cent GFP and per cent cell viability at different WFA concentrations at 24 h pi is shown.

A collection of 66 natural compounds was screened for antiviral activity against CHIKV-GFP in BHK-21 and ERMS cells using the above assay. Following the primary screening, three compounds showed ≥80% CHIKV inhibition and >80% cell viability at 10 μM concentration in both cell lines (Fig 1A, S1 Table). In the secondary screening, these three compounds were tested for antiviral activity at lower concentration ranges (0.1, 0.5, and 1 μM) in BHK-21 and ERMS cells (Fig 1B). The compound no. 24 showed >80% CHIKV inhibition at 1 μM concentration in BHK-21 cells, which was significantly reduced at lower concentrations. The other compounds showed <20% CHIKV inhibition at 1 μM concentration. In EMRS cells, all three compounds showed ~40% or less CHIKV inhibition at lower concentrations. Compound no. 24 was identified as WFA, a steroidal lactone isolated from the medicinal plant *Withania somnifera*. The antiviral dose-response curve of WFA in BHK-21 showed a half maximal inhibitory concentration (IC_50_) of 0.51 μM and a 50% cell cytotoxicity (CC_50_) concentration of 9.95 μM (Fig 1C). The selectivity index (SI) of WFA was calculated as 19.2, thus making it a potential antiviral compound for further evaluation.

### WFA shows antiviral activity against wild-type CHIKV in different cell types

**Fig 2:**
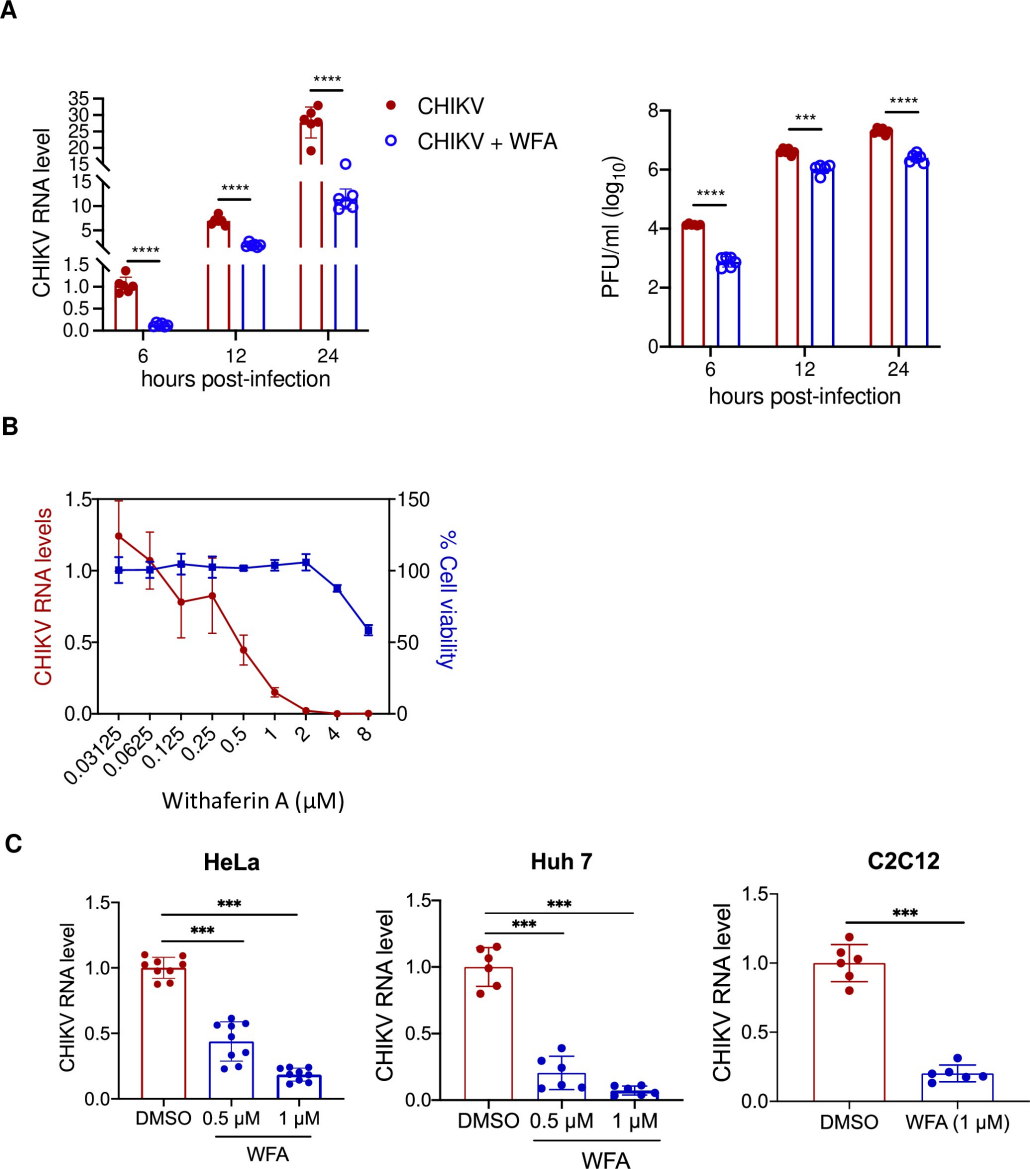
Anti-CHIKV activity of WFA in different cell lines. (A) ERMS cells were infected with CHIKV at 1 MOI. At 0 h pi, the cell culture medium was supplemented with WFA (1 μM) or the vehicle DMSO (control). The total RNA was isolated at different times pi to quantify the intracellular viral RNA levels by qRT-PCR. The relative levels of the CHIKV RNA are shown in the left panel, where the CHIKV RNA level at 6 h pi in the control was taken as 1. The culture supernatant from the CHIKV-infected cells was collected, and the virus titers determined by the plaque assay are shown in the right panel. (B) ERMS cells infected with CHIKV (1 MOI) were treated with different concentrations of WFA. The cells were processed at 6 h pi for the intracellular viral RNA quantitation by qRT-PCR and cell viability by the MTT assay. The line graph demonstrating the relative viral RNA levels and per cent cell viability at the indicated WFA concentrations is shown. The viral RNA levels and per cent cell viability were normalized to the respective vehicle-only controls. (C) HeLa, Huh7, or C2C12 cells were infected with CHIKV (MOI 1) in the presence of WFA or DMSO (vehicle control), and the total RNA was isolated at 6 h pi. The relative CHIKV RNA levels determined by qRT-PCR are presented where the level of CHIKV RNA in the control cells was taken as 1. The student’s t-test was used to calculate the *p* values; *p<0.05, **p<0.01, ***p<0.001.

The screening assay showing the WFA anti-CHIKV activity in BHK-21 and ERMS cells used a recombinant virus based on the LR2006 OPY1 strain of CHIKV. Further on, we tested the WFA antiviral activity using the wild-type CHIKV strain (IND-06-Guj) isolated from an epidemic in India. The mounting evidence has implicated skeletal muscle as an important site in CHIKV disease development [34–37] Therefore, we chose to use ERMS cells of the muscle lineage for further study. To rule out WFA directly interfering with the GFP fluorescence signal, the WFA antiviral activity was studied by measuring the level of CHIKV genomic RNA and extracellular virus titers in the presence or absence of WFA (Fig 2A). CHIKV replicated efficiently in ERMS cells in the absence of WFA. However, virus replication was significantly reduced (*p*<0.001) in the presence of WFA based on the viral RNA levels and virus titers. Compared to the control, the viral RNA levels were 2.5–8 folds lower, and the viral titers were 4–16 folds lower in the presence of WFA at different time points during the virus life cycle. The IC_50_ and CC_50_ values in ERMS cells (Fig 2B) were 0.45 μM and 8.76 μM, respectively, and the SI was 19.4.

We next examined the WFA anti-CHIKV activity in HeLa cells (human epithelial cell line derived from a cancerous tumour of the cervix, adenocarcinoma), Huh7 cells (human hepatocyte-derived cancer cell line), and C2C12 (myoblast line established from the normal adult C3H mouse leg muscle) by measuring the viral RNA in the infected cells at 6 h pi in the presence or absence of WFA. A significant inhibition (50–80%) of CHIKV replication was seen (*p*<0.001) in all of these cell lines when WFA was used at 0.5 μM, and this inhibition was further enhanced (80–90%) at the higher 1 μM concentration of WFA (Fig 2C). These data show that the WFA antiviral activity against CHIKV is not cell type dependent or restricted to a particular cell type.

### WFA shows antiviral activity in the mouse model of CHIKV infection

**Fig 3:**
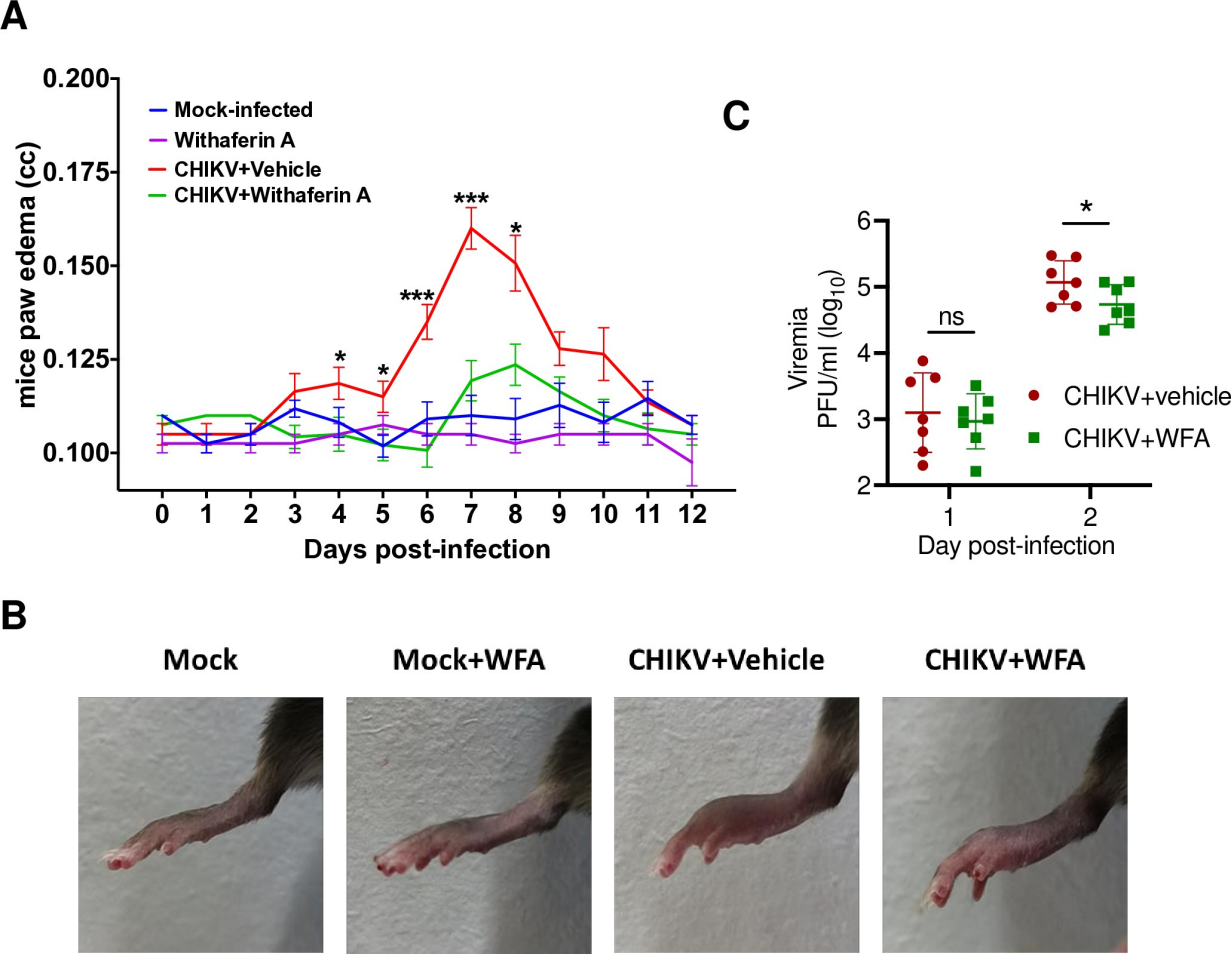
WFA antiviral activity in the mouse model of CHIKV infection. C57BL/6 mice of 12 weeks of age were mock-infected or infected sub-cutaneous with 10^4^ PFU of CHIKV and treated with vehicle alone or WFA (5 mg/kg) given intra-peritoneal twice a day. The first dose of WFA was delivered 4 h pi. The mice in all 4 groups were followed for 2 weeks, and the paw edema was measured daily using a digital plethysmometer. (A) A line graph demonstrating the mouse paw edema on different d pi is presented. The Boneforreni post hoc test, followed by a two-sided independent t-test, was used to calculate the *p* values: *p<0.0332, **p<0.0021, ***p<0.0002, ****p<0.0001. (B) The representative images showing the footpad swelling observed in different treatment groups at 7 d pi are presented. (C) The CHIKV load in the mouse serum at 1 and 2 d pi, as determined by the plaque assay, is shown. The student’s t-test was used to calculate the *p* values: *p<0.05, **p<0.01, ***p<0.001, ns = not significant.

We have established a mouse model of CHIKV infection in adult C57BL/6 mice where CHIKV-infected mice show viremia and swelling of the footpad, which is self-resolving. We tested the antiviral potential of WFA in 12-week-old C57BL/6 mice that were inoculated sub-cutaneous in the foot pad with CHIKV. Mice inoculated with CHIKV started to show footpad swelling at 4–5 days post-infection (d pi), with intense swelling observed at 7 d pi, after which the swelling began to resolve and reached the normal level around 10–12 d pi (Fig 3A and 3B). The CHIKV-infected WFA-treated mice showed none or minimal edema which was statistically not different from that seen in the mock-infected mice. Importantly, edema in the CHIKV-infected WFA-treated mice was significantly reduced (*p*<0.001) compared to that in the CHIKV-infected vehicle-treated mice. Further, we assessed the effect of WFA on viremia by performing plaque assays on serum samples collected on different days. There was no difference in the viral titers in CHIKV-infected WFA-treated mice and CHIKV-infected vehicle-treated mice on 1 d pi; however, on 2 d pi, CHIKV titers were significantly lower (*p*<0.05) by about 50% in WFA-treated mice compared to those in the vehicle-treated CHIKV-infected mice (Fig 3C). No virus was detected in the serum on day 3 and 4 pi. Altogether, these animal data corroborate the WFA antiviral activity against CHIKV seen in the tissue culture cells.

### Antiviral activity of WFA is CHIKV-specific

**Fig 4:**
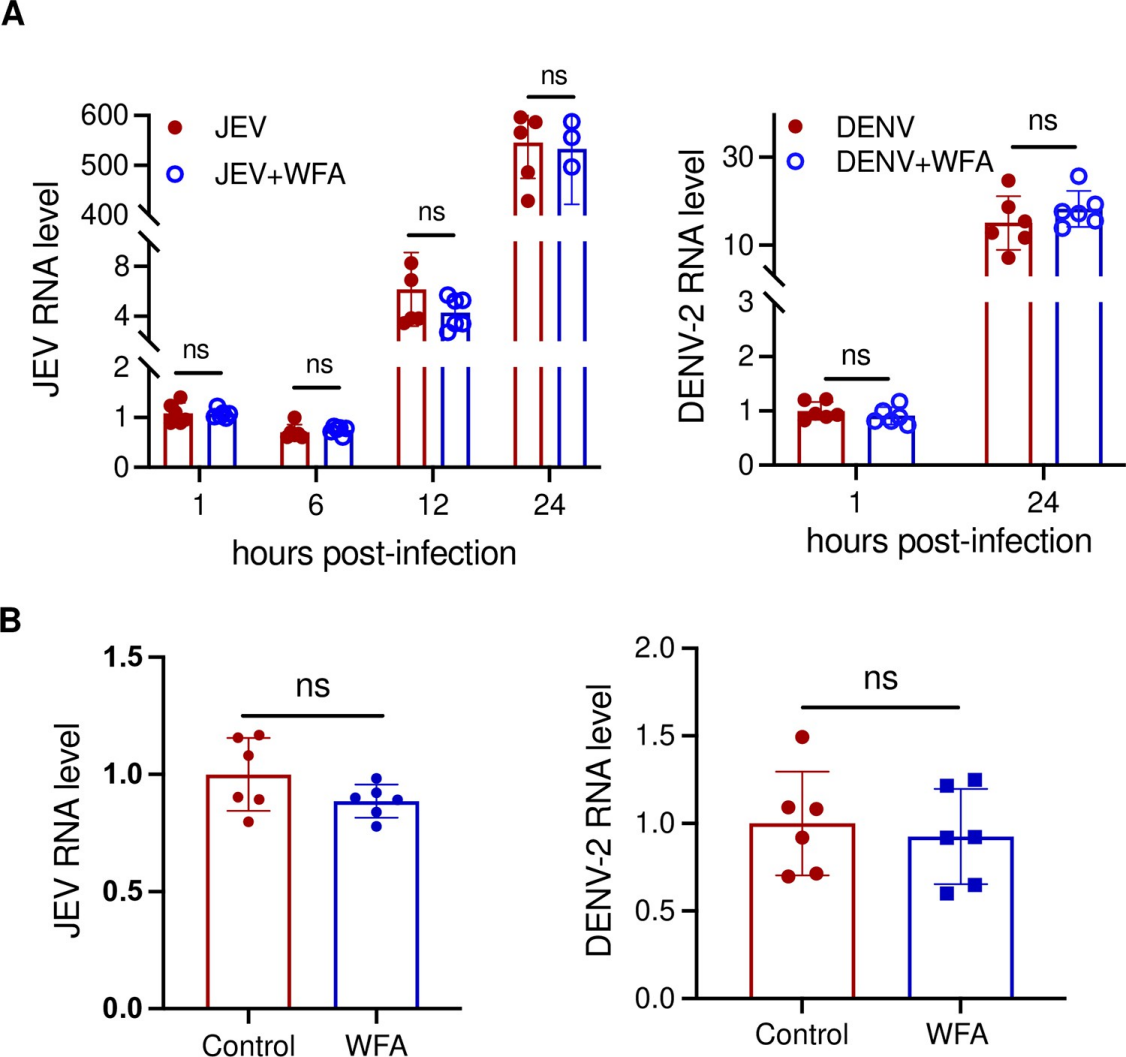
Antiviral activity of WFA is CHIKV specific. (A) ERMS cells were infected with JEV (MOI 1) or DENV-2 (MOI 5) in the presence of 1 μM WFA or DMSO (control). The total RNA was extracted at different times pi, and the relative level of the viral RNA was determined by qRT-PCR. The virus RNA level at 1 h pi in the control cells was taken as 1. (B) HeLa cells were infected with JEV (MOI 1) or DENV-2 (MOI 5) in the presence of 1 μM WFA or DMSO (control). The total RNA was extracted at 24 h pi, and the relative level of viral RNA was determined by qRT-PCR. The virus RNA level at 24 h pi in the control cells was taken as 1. The student’s t-test was used to calculate the *p* values: *p<0.05, **p<0.01, ***p<0.001, ns = not significant.

To examine if WFA had broad antiviral activity, we studied the replication of other enveloped viruses such as Japanese encephalitis virus (JEV) and Dengue virus serotype 2 (DENV-2), by determining the level of genomic RNA in virus-infected cells in the presence or absence of WFA (Fig 4A). No difference was seen in the JEV genomic RNA levels in EMRS cells at 1, 6, 12, and 24 h pi in the absence or presence of 1 μM WFA. Similarly, no difference was seen in DENV-2 replication in EMRS cells at 1 and 24 h pi in the absence or presence of 1 μM WFA (Fig 4A). In HeLa cells also, no difference was seen in the intracellular RNA levels of JEV or DENV-2 at 24 h pi in the absence or presence of WFA at 1 μM concentration (Fig 4B). These data show that the WFA antiviral activity was specific to CHIKV.

### WFA restricts CHIKV replication at the early stage of replication

**Fig 5:**
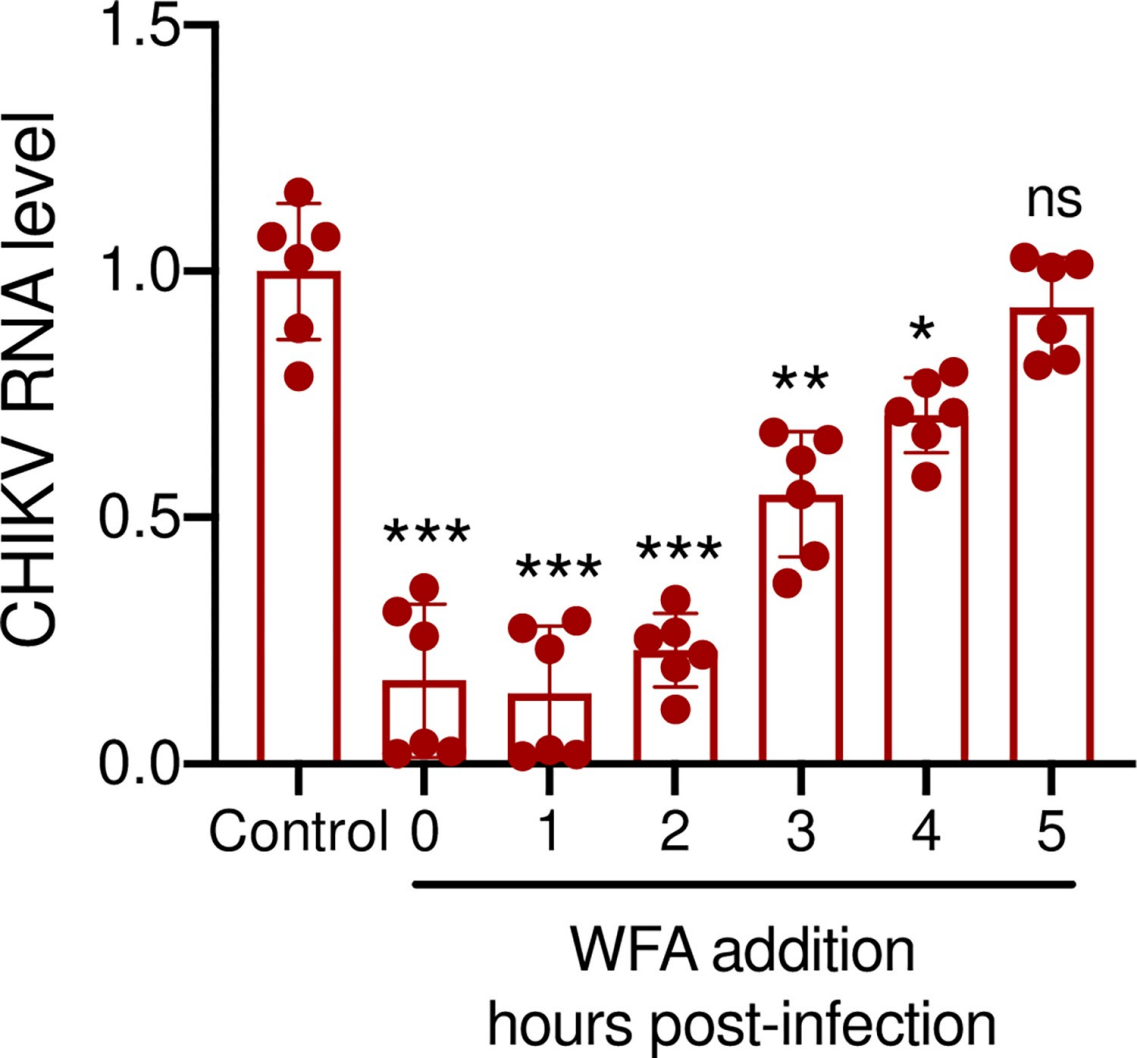
WFA restricts CHIKV replication at the early stage of replication. ERMS cells were infected with CHIKV (1 MOI) and incubated with 1 μM WFA at different time points after the infection. The control CHIKV-infected cells were incubated with DMSO. The cells were harvested at 6 h pi, and the total RNA was extracted. The qRT-PCR was used to determine the CHIKV RNA levels. The relative viral RNA levels are shown. The CHIKV RNA level at 6 h pi in the control cells was taken as 1. The viral RNA level in the control (DMSO-treated) cells was compared with those treated with WFA at different time points. The student’s t-test was used to calculate the *p* values; *p<0.05, **p<0.01, ***p<0.001, ns = not significant.

A successful virus life cycle in a cell involves multiple stages such as receptor binding, entry, viral protein synthesis and genome replication, maturation, and finally, egress. To understand whether WFA affects the early or late stages of CHIKV replication, we performed the time-of-drug-addition assay, where WFA was added to CHIKV-infected cells at different times pi (every hour from 0 to 5 h pi), and the viral RNA levels at 6 h pi were compared to those in the control cells. The WFA treatment given at or before 2 h pi was found to inhibit the viral replication by ~70%, whereas the inhibitory effect was reduced to ~30% to none when the treatment was given at 3 h pi or later (Fig 5). These data suggest that the inhibitory window of WFA action lies early during the infection between 0–2 h pi, which makes the virus entry involving the attachment and uptake, genome uncoating, and viral RNA translation, polyprotein processing as the potential targets of WFA action.

### WFA does not affect CHIKV entry into the cells

**Fig 6:**
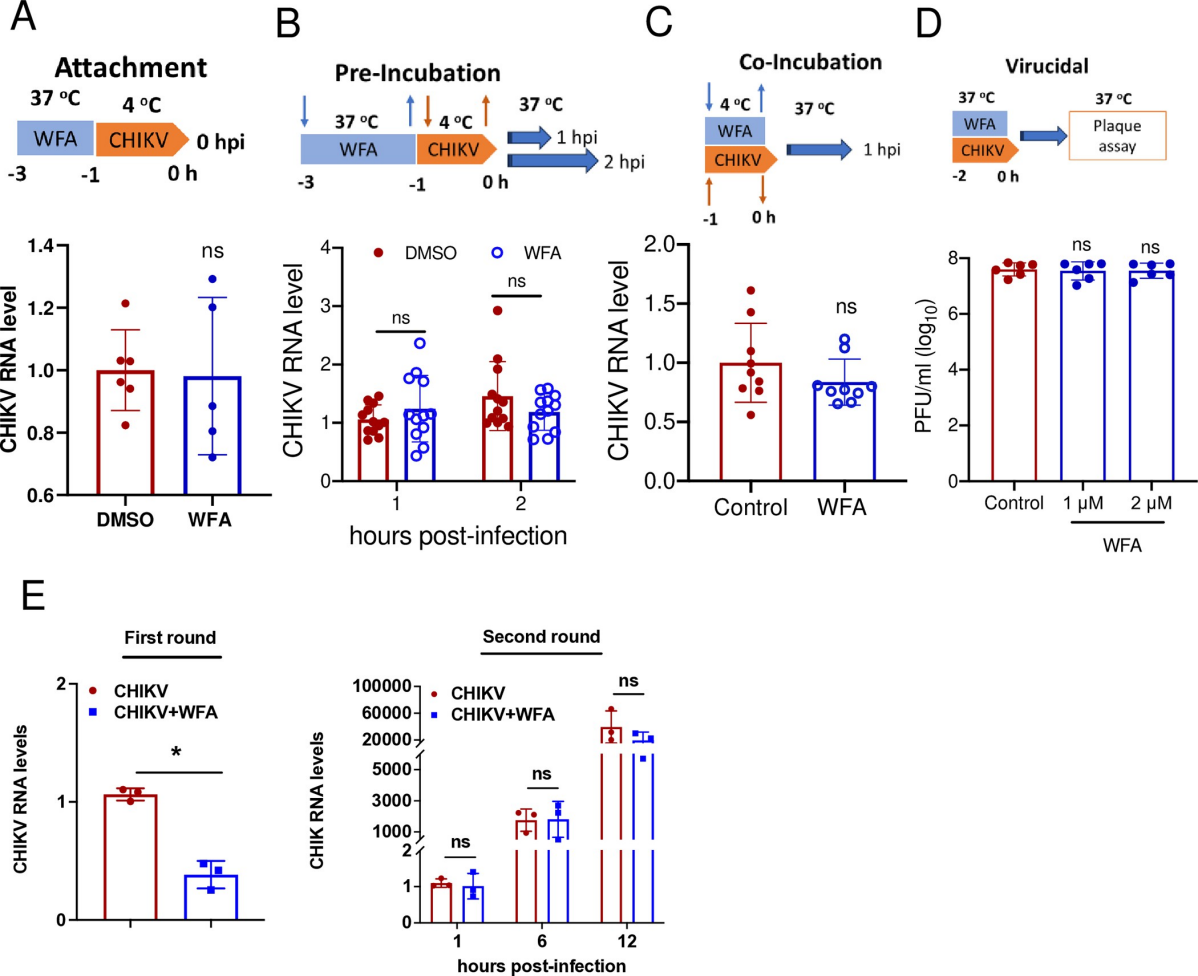
WFA does not affect CHIKV binding and entry into the cells. (A) ERMS cells were incubated with 1 μM WFA or DMSO (as the vehicle control) for 2 h at 37°C. The cells were then incubated for 1 h with CHIKV (MOI 1) on ice to allow the virus attachment but not its uptake. The cells were washed with ice-cold PBS, and the total RNA was extracted. The qRT-PCR was used to determine the level of CHIKV RNA. The relative levels of CHIKV RNA are plotted where CHIKV RNA in the control cells was taken as 1. (B) ERMS cells were incubated with 1 μM WFA or DMSO (as the vehicle control) for 2 h at 37°C. The cells were washed with PBS and then incubated for 1 h with CHIKV (MOI 1) on ice to allow the virus attachment. The cells were incubated for 1 or 2 h at 37°C for viral uptake and then treated with trypsin to remove uninternalized particles. The cells were then washed with PBS, and the total RNA was extracted. The qRT-PCR was used to determine the level of CHIKV RNA. The relative levels of CHIKV RNA are shown where CHIKV RNA in the control cells was taken as 1. (C) ERMS cells were co-incubated with 1 μM WFA and CHIKV (MOI 1) for 1 h at 4°C and incubated for 1h at 37°C for viral uptake. The control cells were incubated with DMSO and CHIKV. The cells were treated with trypsin to remove the uninternalized virion particles. The cells were then washed with PBS, and the total RNA was extracted. The qRT-PCR was used to determine the level of CHIKV RNA. The relative levels of CHIKV RNA are shown where CHIKV RNA in the control cells was taken as 1. (D) For the virucidal assay, CHIKV and WFA (1 or 2 μM) were incubated at 37°C for 2 h. The control had the virus incubated with DMSO. The viral infectivity was determined by the plaque assay. (E) For the first round of infection, ERMS cells were incubated with CHIKV (MOI 0.1) for 1 h. Following this, the cells were incubated with the culture medium supplemented with WFA (1 μM) or the vehicle DMSO (control). The culture supernatant was collected at 12 h pi for determining the virus titers and cells were harvested for the viral RNA quantitation (left panel). For the second round of infection, ERMS cells were infected (MOI 0.1) with the virus collected at 12 h pi from the first round. The culture supernatant and the cells were harvested at different times pi to determine the CHIKV RNA levels (right panel). The student’s t-test was used to calculate the *p* values; *p<0.05, **p<0.01, ***p<0.001, ns = not significant.

The virus entry into the cell involves the virion adsorption or attachment to the receptor on the cell surface, followed by its uptake and delivery into the cytoplasm. To study the attachment of virion particles on the host cell surface, the total RNA was isolated from the control or WFA-pretreated cells after allowing CHIKV binding by incubating the cells with the virus on ice. The qRT-PCR performed to determine the level of the viral RNA showed no significant difference in CHIKV binding to ERMS cells in the presence of WFA compared to the untreated control cells (Fig 6A).

Next, the effect of WFA on CHIKV uptake was studied in ERMS cells pre-incubated or co-incubated with the drug by measuring the intracellular virus RNA level. Any uninternalised virion particles (residual virus outside the cell) were removed by trypsin treatment. The intracellular viral RNA levels assayed at 1 and 2 h pi were unaffected in both pre- and co-incubation conditions compared with the untreated control (Fig 6B and 6C), indicating that WFA did not affect CHIKV uptake into ERMS cells.

To examine if WFA affected the structural integrity of CHIKV, we studied the virucidal property of WFA by determining the infectivity of CHIKV incubated with WFA for 2 h *in vitro*. No difference in virus infectivity (determined as virus titer) was seen in the virus samples incubated with or without WFA (Fig 6D) indicating that WFA did not have virucidal activity against CHIKV.

Next, we examined if CHIKV produced in the presence of WFA had altered infectivity. In the first round of infection, ERMS cells were infected with CHIKV in the presence or absence of WFA and the culture supernatant containing the virus was harvested at 12 h pi. As seen before, CHIKV RNA level in the infected cells was ~60% lower in the presence of WFA compared to that in the control (Fig 6E). To compare the specific infectivity of CHIKV produced in the absence or presence of WFA, CHIKV so produced from the first round was used to infect ERMS cells in the second round at an equal MOI (Fig 6E). No difference was seen in the viral RNA levels at 6 and 12 h pi in cells infected with the virus produced in the presence or absence of WFA in the first round, indicating that WFA did not affect the specific infectivity of CHIKV.

### WFA affects the viral RNA and polyprotein processing early during CHIKV infection

**Fig 7:**
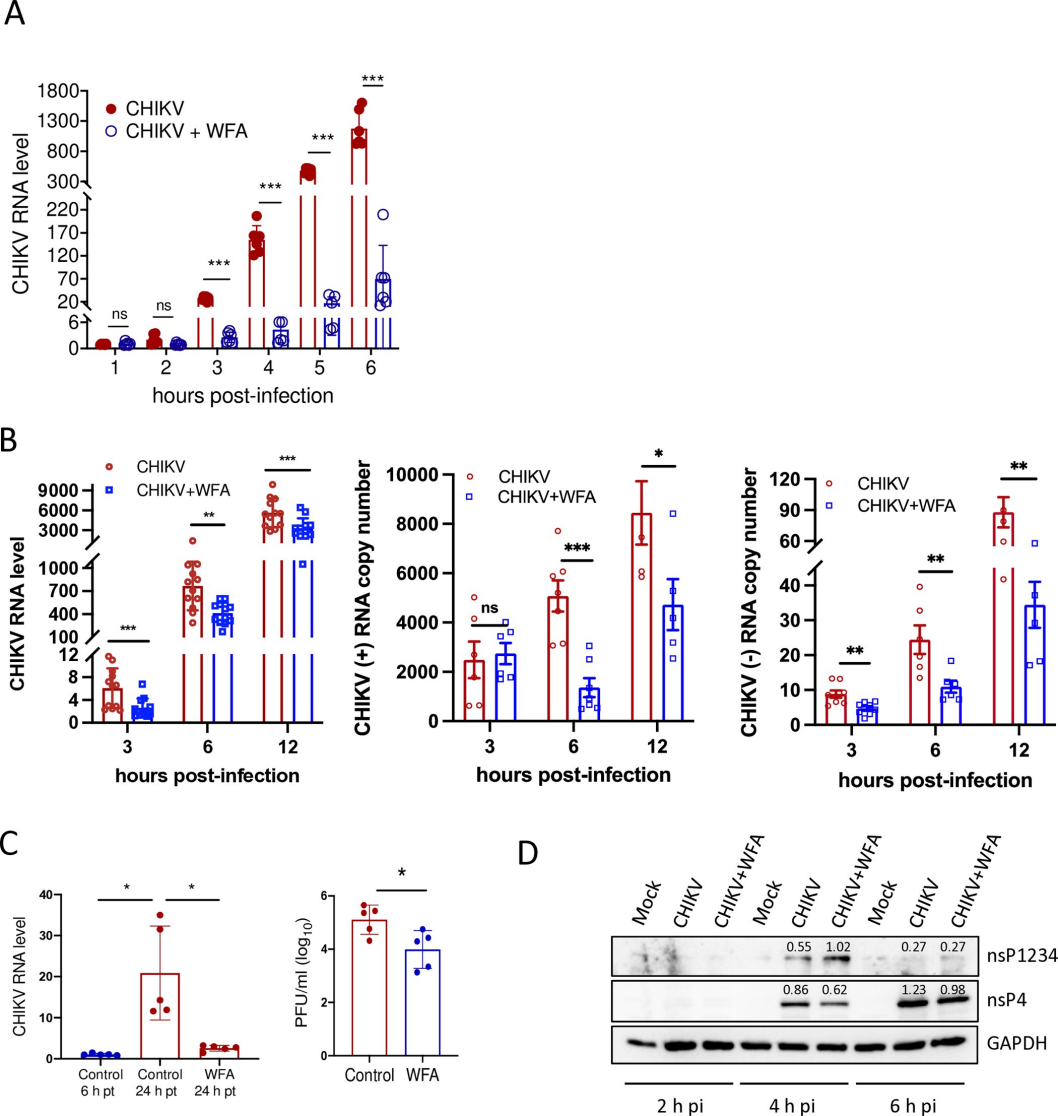
WFA affects CHIKV RNA synthesis and polyprotein processing. (A) ERMS cells were infected with CHIKV (1 MOI) and incubated with 1 μM WFA. The control CHIKV-infected cells were incubated with DMSO. The cells were harvested at different time intervals, and total RNA was extracted. The qRT-PCR was used to determine the CHIKV RNA levels. The relative viral RNA levels are shown. The CHIKV RNA level at 1 h pi in the control cells was taken as 1. (B) ERMS cells were infected with CHIKV (MOI 1) and incubated with 1 μM WFA. The control CHIKV-infected cells were incubated with DMSO. The cells were harvested at different time intervals, and total RNA was extracted. The qRT-PCR was used to determine the CHIKV RNA levels. The relative viral RNA levels are shown in the left panel. The CHIKV RNA level at 1 h pi in the control cells was taken as 1. The CHIKV plus- and minus-sense RNA copy numbers were determined using a standard curve and qRT-PCR, and shown in the middle and right panels, respectively. (C) ERMS cells were transfected with 200 ng of CHIKV RNA, and 6 h later, cells were treated with WFA (1 μM) or DMSO (control). The cells and culture supernatants were harvested 24 h post-transfection (pt) to determine the intracellular CHIKV RNA levels by qRT-PCR (left panel) and the extracellular virus titer by the plaque assay (right panel). The relative RNA levels are plotted where the virus RNA level in the control cells at 6 h pt was taken as 1. (D) ERMS cells were mock infected or infected with CHIKV (1 MOI). The virus-infected cells were incubated with 1 μM WFA. The control CHIKV-infected cells were incubated with DMSO. The cells were harvested at different time points and the cell lysates were western blotted with nsP4 antibody. GAPDH was used as the loading control. The relative band intensity compared to the GAPDH band was measured using the ImageJ software and indicated over the band. The student’s t-test was used to calculate the *p* values; *p<0.05, **p<0.01, ***p<0.001, ns = not significant.

Following the virus uptake and release of the nucleic acid into the cytoplasm, the viral RNA and protein synthesis are the two key steps early during the virus life cycle. We studied the early virus replication by examining the viral RNA levels in the absence or presence of WFA (Fig 7A). No difference in the viral RNA levels was detected at 1 and 2 h pi between the WFA-treated and untreated control cells. However, compared to the control, the WFA-treated cells showed a significantly lower (*p*<0.001) level of viral RNA as early as 3 h pi and this difference was further escalated at the later time points.

The CHIKV genome is a plus-sense RNA and it replicates through a negative-sense RNA intermediate. The above observation of the reduced CHIKV RNA levels in presence of WFA was further validated by determining the copy numbers of CHIKV plus-sense and minus-sense RNAs (Fig 7B). While there was no difference in the plus-sense RNA, the minus-sense RNA synthesis was significantly lagging in the CHIKV-infected WFA-treated cells as early as 3 h pi. Subsequently, the copy numbers of both the plus- and minus-sense RNAs were significantly lower in presence of WFA. As expected, the plus-sense RNA copies were made in far greater numbers than the minus-sense RNA. These data were in conformity with the relative CHIKV RNA estimations made above (Figs 2A and 7A).

To rule out the role of virion binding, endocytosis, and uncoating events in reducing the viral RNA levels seen above, CHIKV RNA replication was studied in ERMS cells transfected with the replication-competent viral RNA in the presence or absence of WFA. A 20-fold increase in the intracellular viral RNA levels was seen in the DMSO-treated control cells at 24 h pt, indicating the successful transfection of RNA and its replication. In the WFA-treated cells at 24 h pt, the viral RNA levels were significantly lower (~10-fold) than in the control cells (Fig 7C). At 24 h pt, a significantly reduced virus titer (~10 fold) was seen in the cells treated with WFA compared to the untreated control cells (Fig 7C). These data suggested that WFA did not affect the virus binding and uptake steps but perhaps targeted the viral protein and RNA synthesis axis early during the CHIKV infection.

The CHIKV nsP4 protein containing the RdRp activity is the key viral protein required for the synthesis of viral genomic RNA. We studied the processing and production of nsP4 in the CHIKV-infected ERMS cells in the presence or absence of WFA. The nsP4 and its precursor nsP1234 proteins could be detected in CHIKV-infected cells as early as 4 h pi. The levels of the nsP4 protein were lower in the WFA-treated CHIKV-infected cells when compared to that in the CHIKV-infected cells not treated with WFA (Fig 7D). Interestingly, the levels of nsP1234, the precursor of nsP4, were higher at 4 h pi in the WFA-treated CHIKV-infected cells than in the untreated cells. Based on the band intensity, at 4 h pi the ratio of nsP1234/nsP4 was 1.56 in WFA-treated CHIK-infected cells whereas it was 0.60 in the CHIKV-infected cells in the absence of WFA. This accumulation of the nsP1234 polyprotein suggested that WFA affected the cleavage of the nsP1234 precursor protein. We did not specifically study the WFA effect on protein synthesis. However, the data here shows no large scale effect on viral protein synthesis.

### Withaferin A binds to CHIKV nsP2 *in silico*

**Fig 8:**
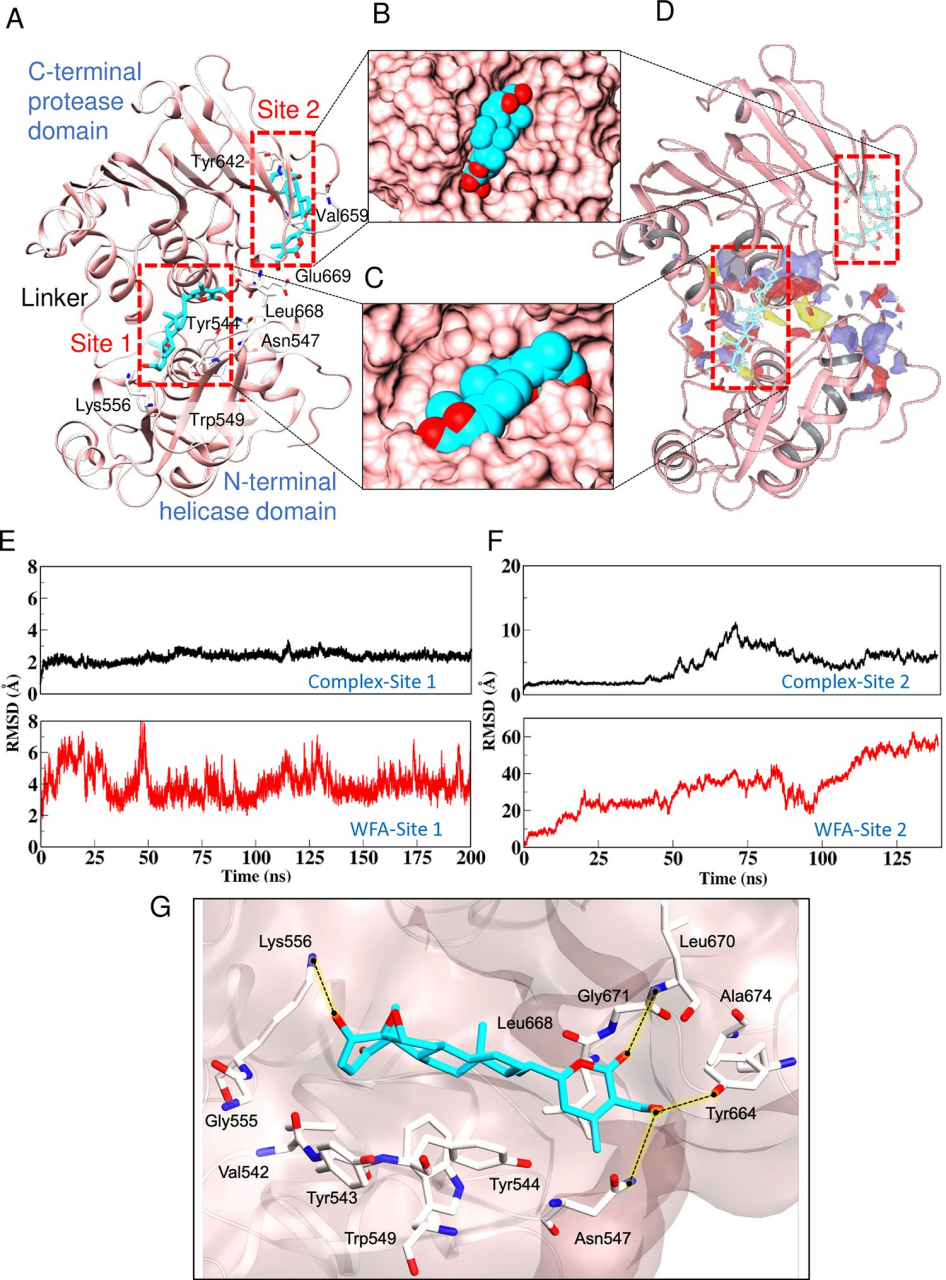
WFA binding to CHIKV nsP2 *in silico*. (A) The CHIKV nsP2 protein is rendered in the cartoon representation and coloured in pink, while WFA is shown in the liquorice representation and coloured atom-wise as C: cyan, and O: red. The binding of WFA at Site 1 and Site 2 is shown. (B-C) The insets show the WFA atomic fitting in the respective pocket; the protein is rendered in the surface view in the pink colour and WFA is shown in the vdW representation. (D) The SiteMap analysis on the CHIKV nsP2 protease. (E-F) The RMSD plots of the WFA-CHIKV complex and WFA at Site 1 and Site 2 from the MD analysis are shown. (G) The interaction map of WFA with the Site 1 residues lining within 4.0 Å is shown. The residues are shown in the liquorice representation and coloured atom-wise as C: white, N: blue, and O: red. The black dotted lines with yellow background represent the hydrogen bonds. The protein is shown in the Quick surf representation.

The above experiments showed that WFA inhibited the CHIKV life cycle early during infection between 1–3 h pi, before the commencement of the virus genome replication, thus making the pre-genome replication steps the main targets of WFA. The RNA genome of CHIKV is translated into a polyprotein, which is subsequently processed to produce the non-structural proteins required for the genome replication. CHIKV non-structural protein 2 (nsP2), is a multifunctional protein with the C-terminal cysteine protease activity [38]. It plays a crucial role in the cleavage of the polyprotein required for producing the crucial viral proteins, including nsP4 with the RdRp activity necessary for replicating the viral genomic RNA [39–41]. To examine if WFA could bind to CHIKV nsP2, we studied their interaction *in silico*. Another member of the withanolide family, Withanone (Wn), that did not show anti-CHIKV activity in the antiviral screening (compound number 26) was included in the study as a negative control. The two most probable binding sites identified by the molecular docking studies are designated as Site 1 and Site 2 (Fig 8A and S3 Table). WFA binds more strongly to Site 1 than Site 2, with docking scores of -8.61 and -4.6, respectively. The negative control Wn had poor docking scores of -3.03 and -2.3 at Sites 1 and 2, respectively. In addition, thermodynamics calculation was performed on docked poses by calculating the MM-GBSA values, which showed WFA’s stronger binding to Site 1 with binding free energy (ΔG_bind_) value as -54.31 kcal/mol, than to Site 2, with ΔG_bind_ value of -34.78 kcal/mol. The overall architecture analysis of the WFA-nsP2 complex indicated that the docked poses were localized adjacent to each other (Fig 8A). However, WFA was well-embedded at Site 1 compared to Site 2 as it had a shallow groove (Fig 8B and 8C). To cross-check the docking outcome in terms of the binding site, an additional analysis was carried out using SiteMap that confirmed Site 1 as the most likely WFA-binding site (Fig 8D and S4 Table). Besides the static analysis, the MD simulations were also carried out to check the conformational stability. The RMSD analysis of the MD trajectories showed that WFA was completely stable at Site 1, and its binding stabilised the overall structure of the nsP2 protein (Fig 8E). On the other hand, WFA exited Site 2 at an early stage (5 ns) and got solvated (Fig 8F). The MD simulation outcomes corroborated the docking findings that WFA could potentially bind to Site 1 on CHIKV nsP2.

To map the key interactions, the interaction fingerprinting identified the amino acid residues contributing to the most stable state obtained from the MD simulation (Fig 8G). WFA was lined by 12 amino acids of nsP2 within 4.0 Å space. The major contributors here included hydrophobic (Val542, Tyr543, Tyr544, Trp549, Tyr664, Leu668, Leu670, Ala674), basic (Lys556) and polar (Asn547) amino acids (Fig 8G). Among these, Asn547, Lys556, Tyr664 and Gly671 established hydrogen bonds with WFA. These data indicate that WFA has potential to interact with the key residues (Asn547, Trp549 and Tyr544) of the substrate binding site/active site of CHIKV nsP2 described by others [42,43].

### Withaferin A binds to CHIKV nsP2 *in vitro*

**Fig 9:**
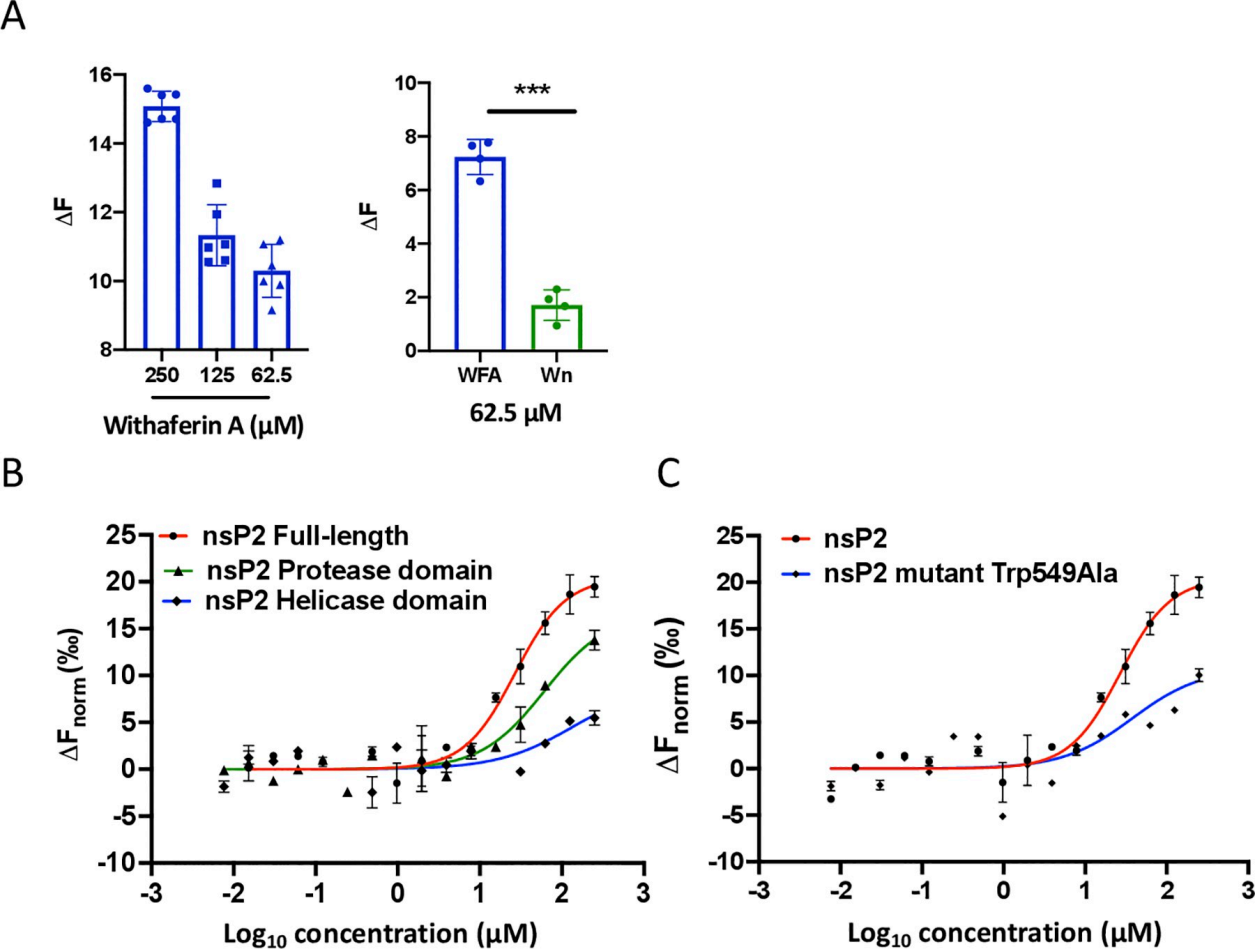
WFA binds to CHIKV nsP2 *in vitro*. (A) Microscale thermophoresis was used to study the binding of the labelled CHIKV nsP2 protein (1 μM) with different concentrations of WFA or Wn. The bar graphs display the fluorescence change (ΔF) obtained on nsP2 binding with different concentrations of WFA or Wn in reference to the unbound protein state in the buffer. The left panel shows the nsP2 binding with different WFA concentrations. The right panel shows nsP2 binding with WFA and Wn at the indicated concentration. The student’s t-test was used to calculate the *p* value; ***p<0.001. (B) To examine the binding and determine the equilibrium dissociation constant between CHIKV nsP2, its protease and helicase domains, and WFA, 1 μM labelled protein was titrated with different concentrations of WFA ranging from 250 to 0.03 μM. (C) To study the effect of the nsP2 mutation on WFA binding 1 μM labelled protein was titrated with different concentrations of WFA. For the binding affinity analysis, ligand-dependent changes in temperature-related intensity change (TRIC) https://nanotempertech.com/nanopedia/tric/are plotted as F_norm_ values against the WFA concentrations in a dose-response curve. The F_norm_ values are plotted as parts per thousand (‰).

We employed microscale thermophoresis (MST) to study the binding of WFA with CHIKV nsP2 experimentally. WFA showed significant binding to CHIKV nsP2 at a range of concentrations (62.5–250 μM) with a fluorescence difference (ΔF) of 10–15 compared to the control (Fig 9A). Importantly, binding of Wn with nsP2 was ~3.5 folds lower than WFA at 62.5 μM concentration (Fig 9A). This is consistent with the molecular docking data where Wn showed weaker binding than WFA to CHIKV nsP2.

The nsP2 protein comprises of the protease and helicase domains. In the next experiment, we studied the binding of WFA with the full-length nsP2, and its protease and helicase domains, using the MST method (Fig 9B). Both the nsP2 protein and the protease domain showed a dose-dependent binding of WFA. While the full-length nsP2 protein and WFA showed significant binding with a K_d_ value of 12 μM, the WFA binding to the isolated protease domain was weaker with a K_d_ value of 64 μM. The WFA binding was very poor or absent with the helicase domain of the CHIKV nsP2 protein.

In order to experimentally validate the WFA binding with nsP2, the computational alanine scanning was carried out on predicted WFA-interacting nsP2 amino acids to identify the residues where substitution with alanine would hinder the WFA binding without affecting the overall native structural conformation of the protein. One such change was identified as the Trp549Ala mutation. The CHIKV nsP2 protein with Trp549Ala mutation showed a significantly reduced binding affinity to WFA as the K_d_ value for the mutant protein was 43 μM compared to the K_d_ value of 12 μM for the native nsP2 (Fig 9C).

Asn547, Trp549 and Tyr544 are involved in the substrate binding site/active site of CHIKV nsP2 [42,43]. Put together, our *in silico* and experimental data demonstrate WFA binding to CHIKV nsP2 in or around the substrate binding site/active site of the protein.

### Withaferin A inhibits the protease activity of CHIKV nsP2

In the early infection stage, nsP1234 polyprotein is cleaved trans by the nsP2 protease into the nsP123 and nsP4 proteins (protease site nsp3/4). Then, the nsP123 is cleaved cis into nsP1 and nsP23 by the nsP2 protease (protease site nsp1/2). Finally, nsP23 is cleaved into nsP2 and nsP3 (protease site nsp2/3) [33]. The nsP3/4 site is rapidly cleaved during infection as the cleavage is absolutely essential for producing nsP4 necessary for the viral RNA synthesis to drive the virus replication. As the antiviral activity of WFA was seen early during the virus life cycle, and the data above showed WFA binding in or around the substrate binding site/active site of the nsP2 protein, we studied the cleavage at the nsP3/4 site by nsP2 in the presence or absence of WFA.

**Fig 10:**
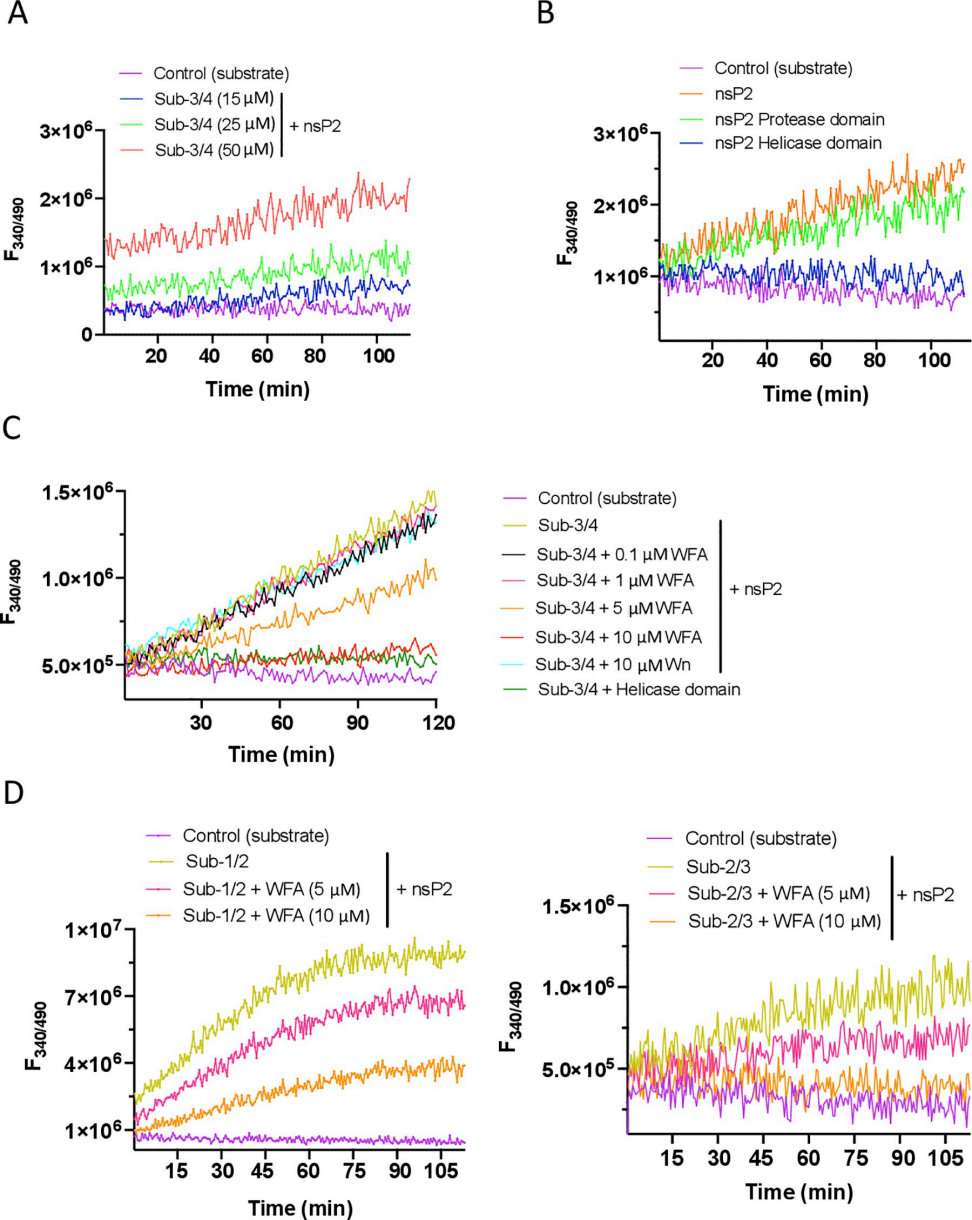
WFA inhibits the protease activity of CHIKV nsP2. A FRET-based protease assay was used to study the nsP2 protease activity. (A) The real-time profile of the proteolytic assay is presented using 1 μM nsP2 protein with different concentrations of the fluorogenic peptide substrate (Sub-3/4). The fluorescence was monitored every 40 sec. (B) The real-time profile of the proteolytic assay is presented using 1 μM each of the nsP2 protein, or its protease and helicase domains with 25 μM fluorogenic peptide substrate Sub-3/4. The fluorescence was monitored every 40 sec. (C) The real-time profile of the proteolytic assay was obtained using different concentrations of WFA or Wn with 1 μM nsP2 and 25 μM peptide substrate Sub-3/4. A proteolytic assay with 1 μM CHIKV nsP2 helicase domain and 25 μM peptide substrate was also performed as the control. The fluorescence was monitored every 30 sec. (D) The real-time fluorescence profile of CHIKV nsP2 (1 μM) protease assay with different concentrations of WFA and 25 μM fluorogenic peptide substrates (Sub-1/2, Sub-2/3). The fluorescence was recorded every 30 sec. For every assay, the background fluorescence of the substrate peptide was monitored without the enzyme and shown as control (substrate).

The CHIKV nsP2 protease assay was performed *in vitro* using the purified nsP2 protein produced in *E*. *coli* and the fluorogenic peptide substrate (Sub-3/4, RAGG↓YIFS) containing the nsP2 protease cleavage site nsP3/4. Following the proteolysis, the fluorogenic peptide is cleaved into two fragments, separating the quencher and fluorophore and increasing the fluorescence with time. To begin with, we demonstrated the protease activity of CHIKV nsP2 with different concentrations of the substrate peptide (Fig 10A). The protein cleaved the substrate peptide efficiently, resulting in an increased fluorescence signal with the increasing substrate concentration, whereas the substrate control reaction without the enzyme did not show any change in the fluorescence. A fluorogenic peptide with randomized sequence (GYFAGSRIS) was not cleaved by the nsP2 protein establishing the substrate specificity of the nsP2 protein activity (S3 Fig).

The nsP2 protein of alphaviruses is a multifunctional enzyme with two key domains: the protease domain and the helicase domain. While the protease domain is involved in the processing of the polyprotein, the helicase domain contains activity involved in RNA unwinding, etc. [44]. We, therefore, validated the above assay with the protease and helicase domains of CHIKV nsP2. The assay clearly showed that the protease domain was able to cleave the substrate efficiently, whereas the helicase domain caused no cleavage (Fig 10B), suggesting that the assay was specific to the CHIKV nsP2 protease activity.

To evaluate the effect of WFA on the nsP2 protease activity, the protease assay was performed in the presence of an increasing concentration of WFA. The cleavage of the substrate peptide by CHIKV nsP2 was significantly inhibited by 5 and 10 μM WFA (Fig 10C). On the contrary, the presence of 10 μM Wn did not affect the nsP2 protease activity and the fluorescence signal was similar to the substrate control reaction (Fig 10C) indicating the specificity of WFA in inhibiting CHIKV nsP2 protease activity. Again, the helicase domain caused no cleavage of the peptide (Fig 10C).

Next, we examined if the cleavage of sites nsP1/2 and nsP2/3 by CHIKV nsP2 was also inhibited by WFA. The peptide substrates representing the cleavage sites nsP1/2 (Sub-1/2, RAGA↓GIIET) and nsP2/3 (Sub-2/3, RAGC↓APSYR), respectively, were cleaved by CHIKV nsP2 (S3 Fig). Importantly, the nsP2 protease activity was inhibited by WFA on both of these substrates (Fig 10D).

WFA could bind the substrate peptides and this could inhibit their cleavage. To examine this, we studied the binding of the substrate peptides with WFA by Isothermal Titration Calorimetry (ITC). While WFA showed binding with CHIKV nsP2, no WFA binding with the substrate peptides used in the protease assay was seen (S4 Fig).

These data show that WFA could inhibit the CHIKV nsP2 protease activity at all the three cleavage sites on the nsP1234 polyprotein produced during the CHIKV replication.

### Reducing agents reverse the WFA-mediated antiviral activity

WFA has two hydrogen bond donors and six hydrogen bond acceptor groups that make it highly reactive and subject to various oxidation patterns, including its ability to target the cysteine sulfhydryl groups of proteins [45–47]. Various studies have shown that the biological effect of WFA gets reversed in the presence of reducing agents such as DTT and NAC [48–62]. According to our computational analysis, WFA is predicted to inhibit CHIKV nsP2 activity by interacting with the key amino acids Asn547, Trp549 and Tyr544, which are critical for substrate recognition [42,43]. Among these, Asn547 could form hydrogen bonds with WFA.

**Fig 11:**
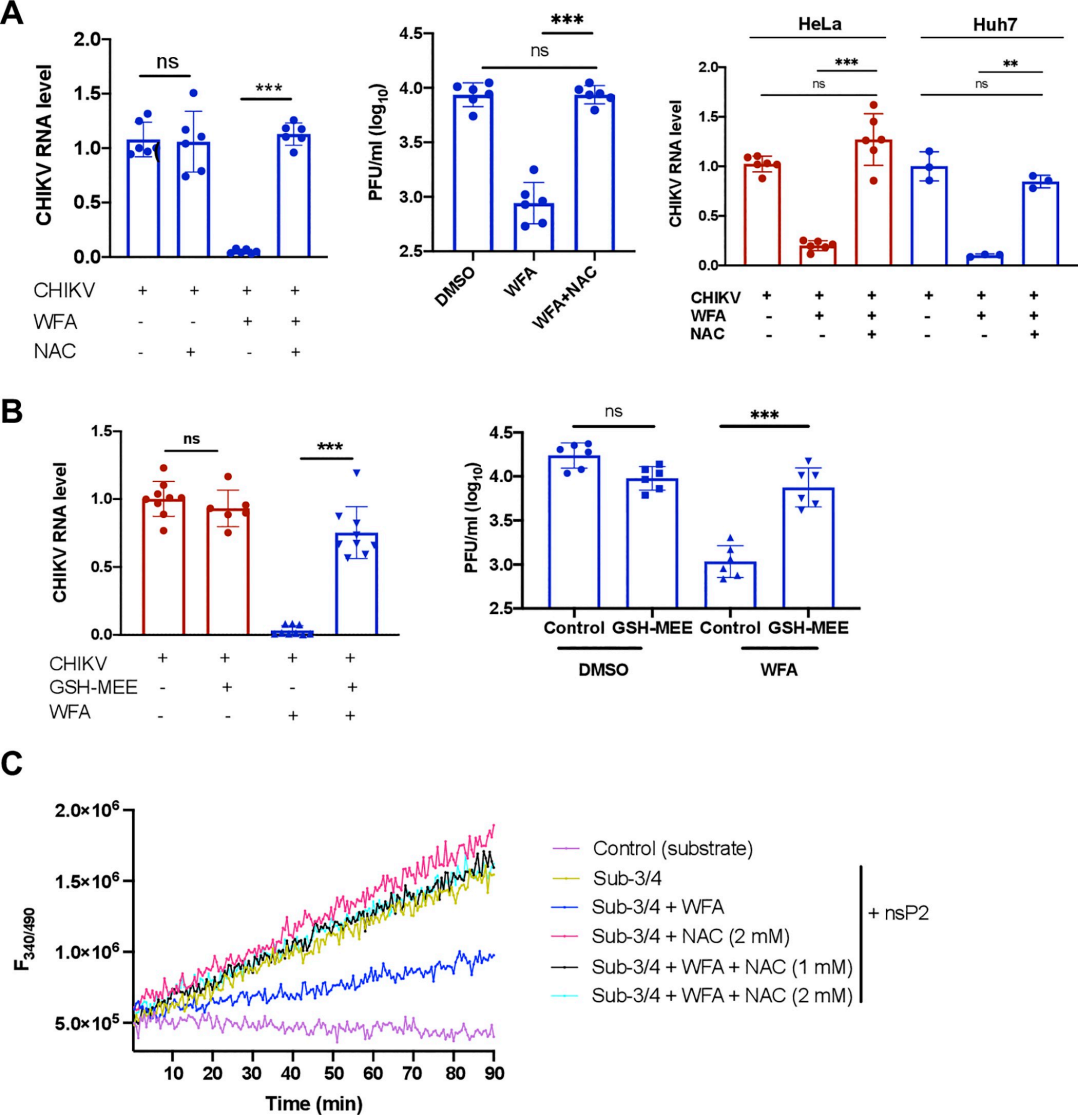
Reducing agents reverse WFA-mediated antiviral activity. (A) ERMS, HeLa or Huh7 cells were infected with CHIKV at 1 MOI. At 0 h pi, cells were incubated with WFA (1 μM) or the vehicle DMSO (control) in the presence or absence of NAC (3 mM) for 6 h. The total RNA isolated from ERMS cells was used to quantify the intracellular viral RNA levels by qRT-PCR (left panel). The culture supernatant from the CHIKV-infected ERMS cells was collected, and the virus titers were determined by the plaque assay (middle panel). The total RNA isolated from HeLa and Huh7 cells was used to quantify the intracellular viral RNA levels by qRT-PCR (right panel). (B) ERMS cells were infected with CHIKV at 1 MOI. At 0 h pi, cells were incubated with WFA (1 μM) or the vehicle DMSO (control) in the presence or absence of GSH-MEE (3 mM) for 6 h. The total RNA isolated from ERMS cells was used to quantify the intracellular viral RNA levels by qRT-PCR (left panel). The culture supernatant from the CHIKV-infected ERMS cells was collected, and the virus titers were determined by the plaque assay (right panel). (C) The real-time fluorescence profile of CHIKV nsP2 (1 μM) protease assay with 5 μM WFA and 25 μM peptide substrate in the presence or absence of NAC at the indicated concentration was recorded every 30 sec. In the control (substrate) assay, the background fluorescence of the substrate peptide was monitored without the enzyme. The student’s t-test was used to calculate the *p* values; *p<0.05, **p<0.01, ***p<0.001, ns = not significant.

To confirm if the oxidation property of WFA was involved in CHIKV nsP2 protease inhibition, we studied CHIKV replication in ERMS cells in the presence of N-acetylcysteine (NAC) (Fig 11A). CHIKV replication was unaffected when treated with NAC alone. As seen before, CHIKV RNA and the virus titers were significantly reduced in the presence of WFA. However, NAC was able to completely reverse the CHIKV inhibitory effect of WFA treatment both at the viral RNA and titer levels. Similar observations on NAC reversing the WFA antiviral effect were made in HeLa and Huh 7 cells infected with CHIKV. Another reducing agent, Glutathione-monoethyl ester (GSH-MEE), showed a similar reversal of the WFA antiviral effect in ERMS cells infected with CHIKV (Fig 11B). The NAC and GSH-MEE concentrations used in these experiments were not toxic to ERMS cells (S5 Fig).

We also studied the effect of NAC on WFA-mediated inhibition of the protease activity of CHIKV nsP2 *in vitro*. Using the nsP2 protease assay as above, we found that CHIKV nsP2 protease activity was inhibited by WFA. Importantly, the presence of NAC in the reaction rescued the CHIKV nsP2 protease activity inhibited by WFA (Fig 11C).

Together, these data show that CHIKV antiviral activity of WFA could be reversed by reducing agents such as NAC and GSH-MEE. This may be related to the abrogation of the WFA binding-mediated inhibition of the CHIKV nsP2 protease activity by these agents.

### WFA inhibits Sindbis virus replication

**Fig 12:**
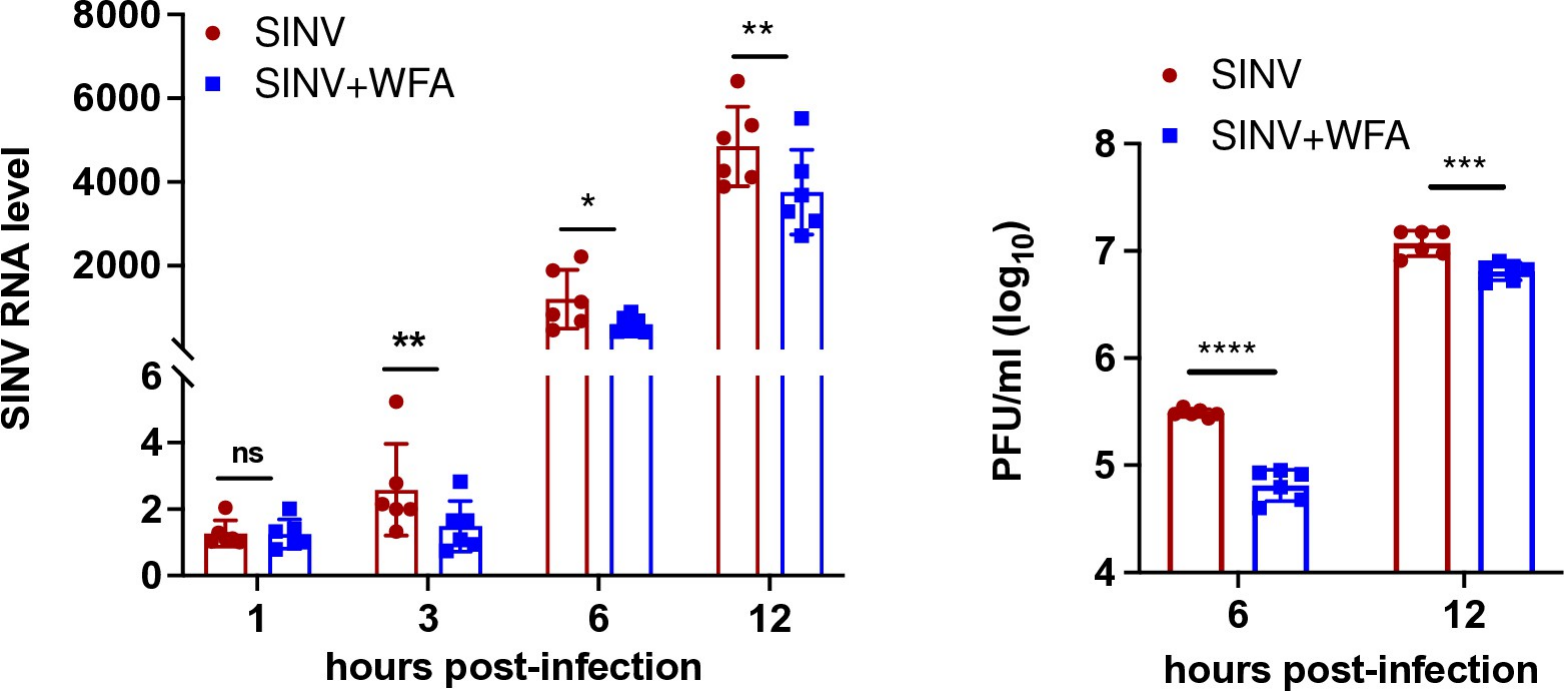
Replication of SINV in presence of WFA. ERMS cells were infected with SINV (MOI 1) in the presence of 1 μM WFA or DMSO (control). The cells and culture supernatants were harvested at different times pi. The total RNA was extracted from the cells, and the relative level of the viral RNA was determined by qRT-PCR. The virus RNA level at 1 h pi in the control cells was taken as 1. The relative viral RNA levels are shown in the left panel. The right panel shows the SINV titers. The viral RNA levels and titers in the control cells were compared with those in the WFA-treated cells. The student’s t-test was used to calculate the *p* values; *p<0.05, **p<0.01, ***p<0.001, ns = not significant.

Sindbis virus (SINV) and CHIKV are both alphaviruses that have a similar genome organization and follow a similar replication strategy. The nsP2 protease is critical to SINV replication in the same manner as in the case of CHIKV. The SINV nsP2 protein has 58% amino acid sequence identity and 74% sequence similarity to the CHIKV nsP2 (S6 Fig). We examined in SINV the conservation of the ten amino acids predicted above *in silico* to be important for WFA binding to CHIKV nsP2. Of these, four amino acids were conserved and three showed conservative substitution between the CHIKV and SINV nsP2 proteins (S6 Fig). In light of this, we studied if WFA inhibited the SINV replication (Fig 12). Similar to CHIKV, WFA inhibited SINV replication early during replication as seen by the reduced viral RNA levels in the SINV-infected ERMS cells. The SINV titers, measured at 6 and 12 h pi (Fig 12), were also significantly reduced in the WFA-treated cells compared to the control.

## Discussion

CHIKV is a re-emerging alphavirus endemic to tropical and subtropical areas. The virus has already affected more than 100 countries worldwide and constitutes a risk to many other non-endemic countries where its transmission vector, *i*.*e*. *Aedes* mosquito, is established. This makes CHIKV antiviral discovery a high priority. In this study, we screened a collection of natural molecules for their potential to inhibit CHIKV replication. We identified WFA showing robust CHIKV inhibition in multiple cell lines with a high selectivity index of ~19.

WFA is a steroidal lactone, one of the biologically active chemical constituents of the medicinal herb *Withania somnifera*, commonly known as Ashwagandha. *W*. *Somnifera* is an important ayurvedic medicine, that along with its active phytoconstituents has demonstrated immunomodulatory, anti-inflammatory [63,64], anti-tumorogenic [65,66], antiviral [67–73], antifungal [74] and antibacterial [49,75–77] properties. A *W*. *Somnifera*-based Indian traditional formulation (Amukkara Choornam) was reported to alleviate arthralgia and clear the virus from the brain and joint tissues in CHIKV-infected C57BL/6J mice [70]. Besides WFA, our collection of natural compounds had several phytoconstituents of *W*. *Somnifera*, such as Withanoside (IV and V), Withanolide (A and B), Withanostraminolide -12 deoxy, Methoxy-withaferin A, Quercetin glucoside, and Withanone (Wn). Importantly, except WFA, none of these showed any significant anti-CHIKV activity in BHK-21 and ERMS cells (Compound nos. 25, 26, 50,51, 52, 53, 54 in Fig 1 and S1 Table).

The steroidal lactones such as WFA or Wn have been studied against various viruses such as HPV [78], IAV [79,80], HCV [81], HSV [72], HIV [69] and SARS-CoV2 [82] and these were shown to target the viral protein activity to exert the antiviral action. Their antiviral activity has been studied against SARS-CoV-2 [82–86], and recently, *W*. *somnifera* was approved for clinical trials against COVID-19 [83]. Research groups have independently demonstrated WFA binding to the spike protein of SARS-CoV-2, thus inhibiting virus binding and entry into the host cells [87–90]. Wn and WFA were also predicted to interact with the host transmembrane protease serine 2 (TMPRSS2) and block SARS-CoV-2 entry into the cells [91].

Our study shows that WFA specifically targets CHIKV and does not affect the replication of other enveloped viruses outside the Alphavirus genus, such as the Japanese encephalitis and Dengue viruses. We show that WFA does not affect the CHIKV infectivity nor interfere with the virus binding and uptake into the host cells. Importantly, WFA was seen to block the CHIKV life cycle at the early time of infection between 1–3 h pi, before the commencement of the virus genome replication, hence making the pre-genome replication steps the main targets of WFA. Furthermore, the WFA-mediated inhibition of the replication of exogenously-transfected viral RNA indicates that WFA targets the post-entry translation/replication axis of the CHIKV life cycle, bypassing the virion binding, endocytosis, and uncoating events. The alphavirus genome replication requires the translation of the incoming genomic RNA into a polyprotein, which is subsequently cleaved into several viral proteins by the viral protease [9,92]. Interestingly, several *in silico* studies have predicted that WFA or Wn interacts with the viral proteases such as HIV-1 protease [69] and SARS-CoV-2 main proteases [89,93–97] with high affinity and may inhibit the virus replication. Recently, WFA and Wn were demonstrated as the covalent inhibitors of the SARS-CoV-2 Main Protease activity [93].

The CHIKV non-structural protein (nsP2) is a multifunctional protein, with its C-terminal half containing the proteolytic domain nsP2 is considered a cysteine protease. The nsP2 proteolytic activity is crucial for the cleavage of the non-structural polyprotein precursor (nsP1234) into different non-structural proteins that form the viral replication complex, thus making it the most attractive target for the development of antivirals against CHIKV. The structural elucidation of CHIKV nsP2pro (PDB ID: 3TRK, 4ZTB) has revealed that the highly conserved Cys478 and His548 residues (the numbers of amino acid residues correspond to their numbers in the CHIKV nsP2) constitute the active site catalytic dyad crucial for its protease activity [31,39]. The CHIKV nsP2 protease active site orientation of Cys478 and His548 is similar to the catalytic dyad of papain (PDB ID: 1PPN). CHIKV nsP2pro has 40% sequence identity with Venezuelan Equine Encephalitis virus (VEEV) nsP2pro (PDB ID: 2HWK) and 44% sequence identity with Sindbis virus (SINV) nsP2pro (PDB ID: 4GUA) [98]. The 3D structure of alphavirus proteases suggests that the catalytic dyad of CHIKV nsP2 protease corresponds to nsP2 catalytic dyad residues Cys481 and His558 in SINV (SINV; PDB ID: 4GUA), Cys477 and His546 (PDB ID: 2WHK) in VEEV [98,99] and Cys478 and His548 residues in Semliki Forest virus (SFV). Mutation of these residues resulted in complete loss of the protease activity [31,39,100,101]. Given the critical role of CHIKV nsP2 protease in the virus life cycle, several studies have employed a computer-aided drug design approach of molecular docking and molecular dynamics simulations on the crystal structure of CHIKV nsP2 protease to identify potential CHIKV inhibitors [14,20,102–108].

Our *in silico* studies suggested that WFA could form a hydrogen bond with Asn547, a conserved residue in the nsP2 protease across alphaviruses, that is reported to be a critical substrate binding residue [42,43]. The Asn547Ala mutation established the functional role of Asn547 in the substrate specificity in CHIKV nsP2 protease. Another set of nsP2 residues in our data, lined within 4 Å of WFA with hydrophobic interaction, Tyr544, Trp549, and Leu670, have been reported to be crucial for substrate recognition and binding [42]. Our MST studies showed that the Trp549Ala mutation reduced the WFA binding to the nsP2 protein, although its effect on WFA sensitivity of the virus in the cell culture remains to be shown. Altogether, these observations indicated that WFA most likely blocked the binding of the substrate. Indeed, using the MST method, we showed WFA binding to CHIKV nsP2 in a dose-dependent manner with the K_d_ value of 12 μM. Importantly, CHKV nsP2 protease activity was inhibited by WFA in a dose-dependent manner, as shown in the FRET-based proteolytic activity assay. The mutant data thus validate the MST findings and the *in silico* prediction on WFA binding with CHIKV nsP2 protein. Passaging the virus in presence of WFA might generate WFA-resistant virus mutants. These could be useful in further validating the WFA binding site on nsP2.

We studied the effect of reducing agents (NAC and GSH-MEE) on WFA-mediated CHIKV inhibition in the cell culture system. We found that these reducing agents could completely reverse the CHIKV inhibitory effect of WFA treatment both at the viral RNA and titer levels. These results indicate that WFA targets the CHIKV nsP2 protease and inhibits its enzymatic activity by its oxidation potential.

The CHIKV antiviral activity seen in the cell culture was replicated in the C57BL/6 mouse model of CHIKV infection. In CHIKV-infected, WFA-treated mice, we observed a significant reduction in viremia by ~50% compared to the vehicle control group. We found that the WFA treatment significantly reduced the morbidity (paw swelling) caused by CHIKV in the mouse model. In addition to WFA inhibiting the virus replication resulting in the lowering of viremia, we cannot rule out the possibility of WFA affecting the CHIKV-induced paw swelling through an indirect route independent of the virus replication. Since early stage of replication was affected in the presence of WFA and we noted the accumulation of the nsP1234 polyprotein, we decided to focus our study on CHIKV nsP2 protein. The effect of WFA on other viral and cellular proteins involved in CHIKV infection, if any, cannot be ruled out. Nonetheless, our data support further investigations on using WFA or its derivatives for the pre-clinical and clinical evaluations to develop novel CHIKV antivirals.

## Supporting information

S1 Fig Infection of BHK-21, Vero, and ERMS cells by CHIKV. (A) The cells seeded overnight were infected with CHIKV-LR-5’GFP as described in the methods. The cell nuclei were stained with the Hoechst stain. Following the nuclei staining, the images were acquired with channels for Hoechst (blue) and GFP (green) using the ImageXpress High-Content Imaging system (Molecular Devices). The representative images are shown. (B) BHK-21, Vero, and ERMS cells seeded overnight were infected with CHIKV at MOI 1. The cells were harvested at different times pi and the total RNA isolated. The CHIKV positive-sense RNA copy numbers determined by qRT-PCR are shown at different times pi.
(TIFF)

S2 Fig Expression and purification of CHIKV nsP2 protein and its protease and helicase domains. *E*. *coli* was transformed with the expression plasmids and the protein purified as described in the Methods. Shown above is the image of a 10% SDS-PAGE of the purified proteins. The molecular weight markers (in kDa) are identified at the left.
(TIFF)

S3 Fig Cleavage of different protease sites in the CHIKV polyprotein. A FRET-based protease assay was used to study the nsP2 protease activity on the cleavage sites nsP1/2 and nsP2/3 and random sequence peptide (negative control). The real-time profile of the proteolytic assay is presented using 1 μM nsP2 protein with different concentrations of the fluorogenic peptide substrates as indicated. In the substrate control assays, the background fluorescence of the peptide substrate was monitored without the enzyme.
(TIFF)

S4 Fig Studies on binding of the peptide substrates with WFA. Isothermal titration calorimetry (ITC) was used to study the binding of the peptide substrates Sub-1/2, Sub-2/3, and Sub-3/4 with WFA. The binding reactions were titrated using 10 μM substrate and 100 μM WFA. The thermograms (top) and fitted binding isotherms (bottom) for the binding of the substrates with WFA are shown. In a control experiment, CHIKV nsP2 (10 μM) binding was titrated with WFA (100 μM). Malvern’s Origin 7.0 Microcal-ITC200 analysis software was applied to obtain the thermodynamic parameter ΔH.
(TIFF)

S5 Fig Cytotoxicity of NAC and GSH-MEE. ERMS cells were treated with different concentrations of NAC and GSH-MEE for 6 h. The cells were harvested, and the MTT assay was performed to calculate the percentage of cell viability.
(TIFF)

S6 Fig Alignment of CHIKV and SINV nsP2 protein sequences. The amino acid sequence of the CHIKV nsP2 protein (GenBank accession: ADZ47896.1) was aligned with the SINV nsP2 protein (GenBank accession: NP_740671) using Blastp (protein-protein BLAST). The CHIKV nsP2 amino acids predicted to be involved in the WFA binding are shown in red. Of these, amino acids conserved between CHIKV and SINV nsP2 are marked with long vertical green box. The conservative amino acid substitutions are marked with short vertical green boxes. Conservative amino acid changes refer to substitutions that replace an amino acid with another having similar properties such as the charge, size and shape, polarity, and hydrophobicity.
(TIFF)

S1 Table Antiviral activity of the natural compounds. The MTT-based cell viability assay was performed using 85 compounds (derived from herbs used in traditional home medicine systems) and 66 compounds were selected that were not toxic at 0.5 to 1 μM concentration using human breast cancer (MCF7) and bone cancer (U2OS) cells, both possessing wild type p53 (similar to the normal cells). All compounds were procured in their purified form (95% purity) from commercial suppliers such as Sigma, Wako and Tokiwa phytochemicals, Japan. The table above shows the anti-CHIKV activity of those 66 compounds in ERMS and BHK-21 cells.
(DOCX)

S2 Table Primers used in the CHIKV strand-specific qRT-PCR. (DOCX)

S3 Table The selection of residues at Site 1 and Site 2 for grid generation. (DOCX)

S4 Table SiteMap analysis highlights Site 1 as the potential WFA binding site. (DOCX)
