# Supplementary figures and images for: Withaferin A inhibits Chikungunya virus nsP2 protease and shows antiviral activity in the cell culture and mouse model of virus infection
Source: PLoS Pathog. 2024 Dec 30;20(12):e1012816. doi: 10.1371/journal.ppat.1012816 (PMC11723598; doi:10.1371/journal.ppat.1012816)

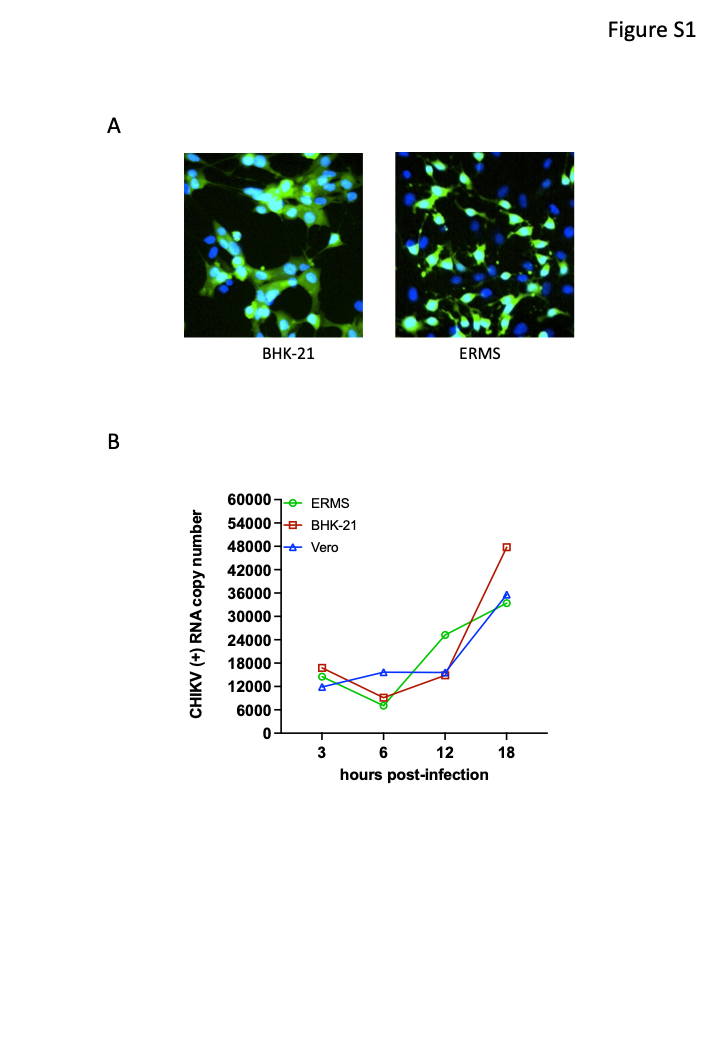

Supplement: S1 Fig — (A) The cells seeded overnight were infected with CHIKV-LR-5’GFP as described in the methods. The cell nuclei were stained with the Hoechst stain. Following the nuclei staining, the images were acquired with channels for Hoechst (blue) and GFP (green) using the ImageXpress High-Content Imaging system (Molecular Devices). The representative images are shown. (B) BHK-21, Vero, and ERMS cells seeded overnight were infected with CHIKV at MOI 1. The cells were harvested at different times pi and the total RNA isolated. The CHIKV positive-sense RNA copy numbers determined by qRT-PCR are shown at different times pi. (TIFF) [file ppat.1012816.s001.tiff]

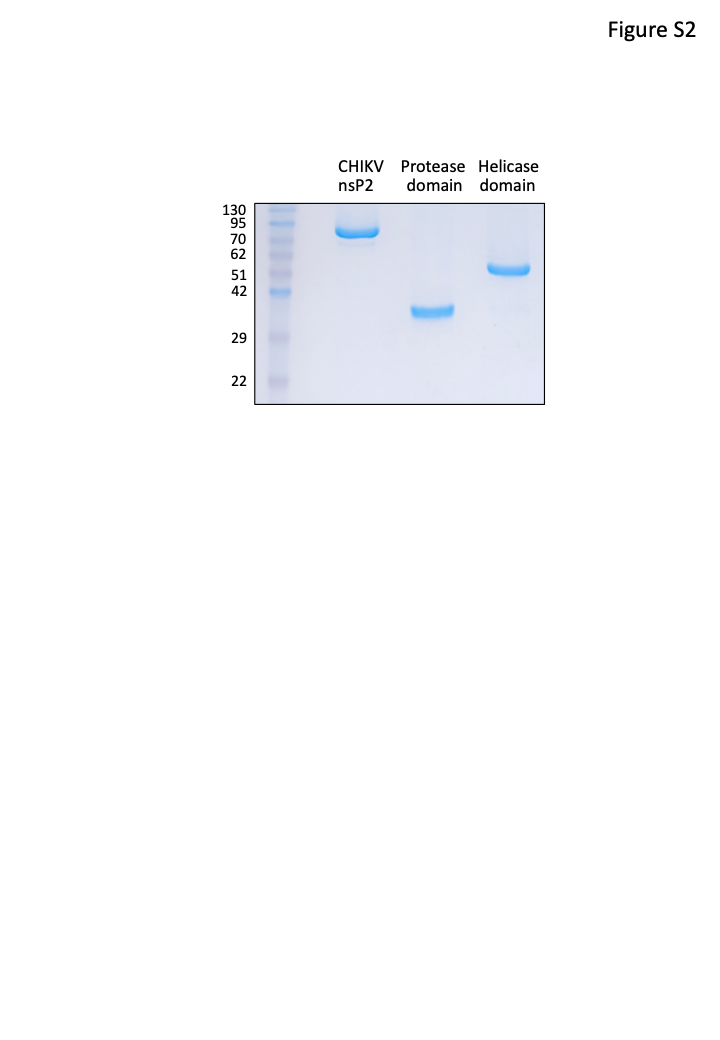

Supplement: S2 Fig — E. coli was transformed with the expression plasmids and the protein purified as described in the Methods. Shown above is the image of a 10% SDS-PAGE of the purified proteins. The molecular weight markers (in kDa) are identified at the left. (TIFF) [file ppat.1012816.s002.tiff]

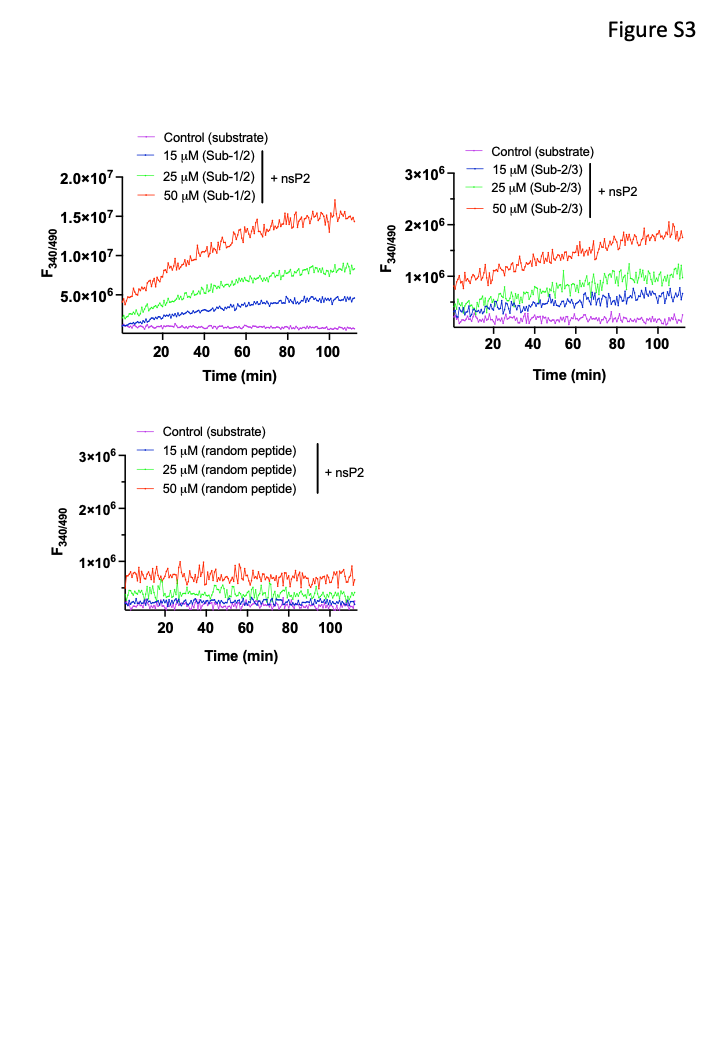

Supplement: S3 Fig — A FRET-based protease assay was used to study the nsP2 protease activity on the cleavage sites nsP1/2 and nsP2/3 and random sequence peptide (negative control). The real-time profile of the proteolytic assay is presented using 1 μM nsP2 protein with different concentrations of the fluorogenic peptide substrates as indicated. In the substrate control assays, the background fluorescence of the peptide substrate was monitored without the enzyme. (TIFF) [file ppat.1012816.s003.tiff]

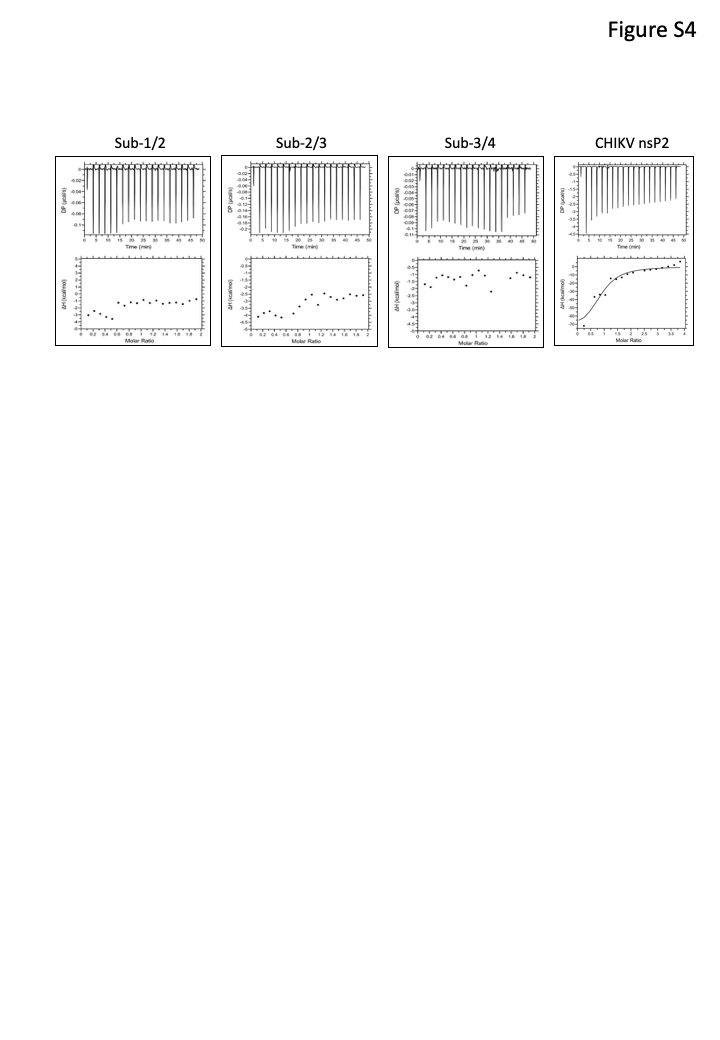

Supplement: S4 Fig — Isothermal titration calorimetry (ITC) was used to study the binding of the peptide substrates Sub-1/2, Sub-2/3, and Sub-3/4 with WFA. The binding reactions were titrated using 10 μM substrate and 100 μM WFA. The thermograms (top) and fitted binding isotherms (bottom) for the binding of the substrates with WFA are shown. In a control experiment, CHIKV nsP2 (10 μM) binding was titrated with WFA (100 μM). Malvern’s Origin 7.0 Microcal-ITC200 analysis software was applied to obtain the thermodynamic parameter ΔH. (TIFF) [file ppat.1012816.s004.tiff]

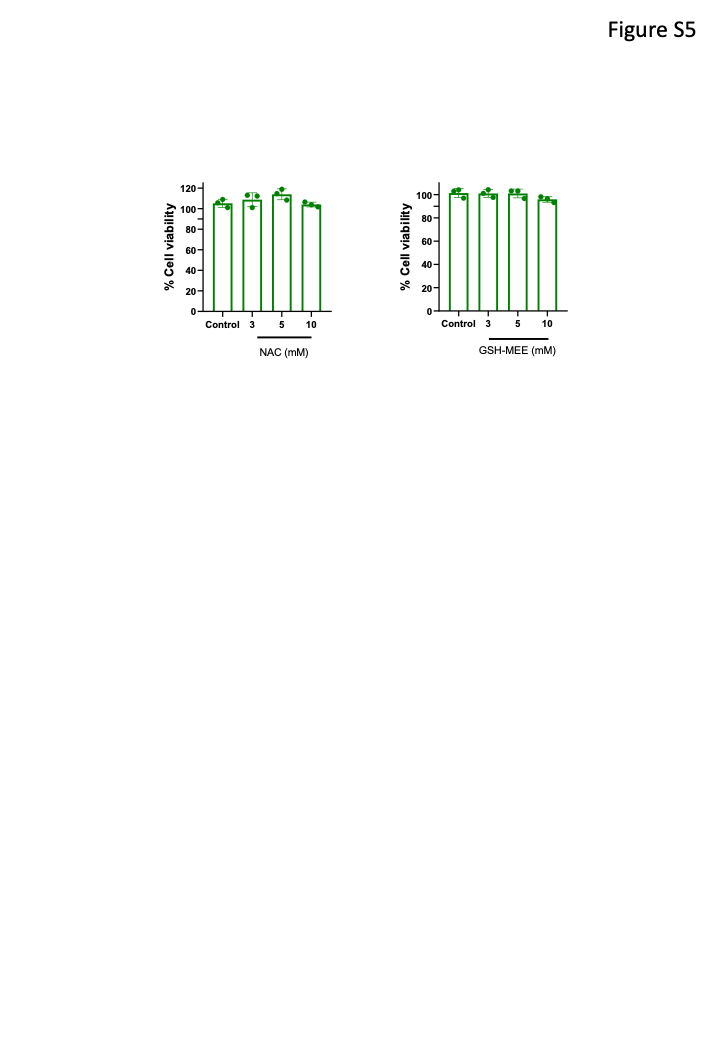

Supplement: S5 Fig — ERMS cells were treated with different concentrations of NAC and GSH-MEE for 6 h. The cells were harvested, and the MTT assay was performed to calculate the percentage of cell viability. (TIFF) [file ppat.1012816.s005.tiff]

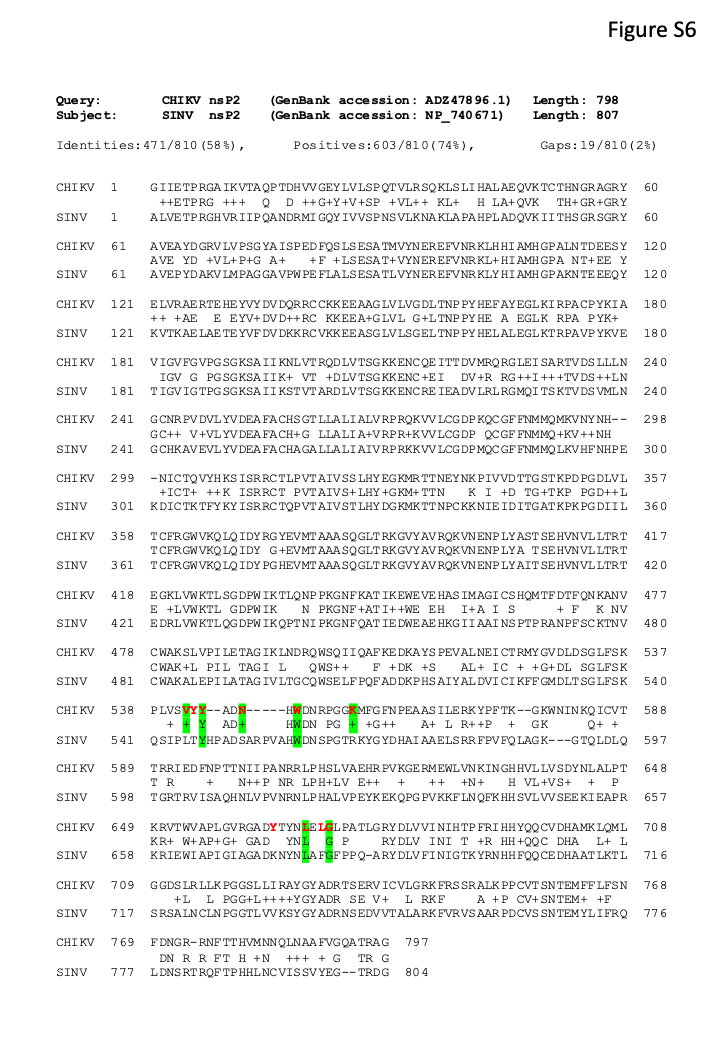

Supplement: S6 Fig — The amino acid sequence of the CHIKV nsP2 protein (GenBank accession: ADZ47896.1) was aligned with the SINV nsP2 protein (GenBank accession: NP_740671) using Blastp (protein-protein BLAST). The CHIKV nsP2 amino acids predicted to be involved in the WFA binding are shown in red. Of these, amino acids conserved between CHIKV and SINV nsP2 are marked with long vertical green box. The conservative amino acid substitutions are marked with short vertical green boxes. Conservative amino acid changes refer to substitutions that replace an amino acid with another having similar properties such as the charge, size and shape, polarity, and hydrophobicity. (TIFF) [file ppat.1012816.s006.tiff]
