# Supplementary material for: Withaferin A inhibits Chikungunya virus nsP2 protease and shows antiviral activity in the cell culture and mouse model of virus infection
Source: PLoS Pathog. 2024 Dec 30;20(12):e1012816. doi: 10.1371/journal.ppat.1012816 (PMC11723598; doi:10.1371/journal.ppat.1012816)
Supplement: S1 Table — The MTT-based cell viability assay was performed using 85 compounds (derived from herbs used in traditional home medicine systems) and 66 compounds were selected that were not toxic at 0.5 to 1 μM concentration using human breast cancer (MCF7) and bone cancer (U2OS) cells, both possessing wild type p53 (similar to the normal cells). All compounds were procured in their purified form (95% purity) from commercial suppliers such as Sigma, Wako and Tokiwa phytochemicals, Japan. The table above shows the anti-CHIKV activity of those 66 compounds in ERMS and BHK-21 cells. (DOCX) [file ppat.1012816.s007.docx]

**Table S1: Antiviral activity of the natural compounds.**

The MTT-based cell viability assay was performed using 85 compounds (derived from herbs used in traditional home medicine systems) and 66 compounds were selected that were not toxic at 0.5 to 1 µM concentration using human breast cancer (MCF7) and bone cancer (U2OS) cells, both possessing wild type p53 (similar to the normal cells). All compounds were procured in their purified form (95 % purity) from commercial suppliers such as Sigma, Wako and Tokiwa phytochemicals, Japan. The table above shows the anti-CHIKV activity of those 66 compounds in ERMS and BHK-21 cell
