# Supplementary material for: Withaferin A inhibits Chikungunya virus nsP2 protease and shows antiviral activity in the cell culture and mouse model of virus infection
Source: PLoS Pathog. 2024 Dec 30;20(12):e1012816. doi: 10.1371/journal.ppat.1012816 (PMC11723598; doi:10.1371/journal.ppat.1012816)
Supplement: S2 Table — (DOCX) [file ppat.1012816.s008.docx]

**Table S2. Primers used in the CHIKV strand-specific qRT-PCR.**

| **Primer** | **Nucleotide Sequence (5'-3')** | **Amplification product** |
| --- | --- | --- |
| Primers for generation of plus- and minus-sense CHIKV RNAs (underlined is T7 polymerase promoter sequence followed by CHIKV nsP2 sequence) | | |
| CHIKp-For | CGTAATACGACTCACTATAGGGGAATAATAGAGACTCCGAGAGG | Positive strand |
| CHIKp-Rev | ACATCCTGCTCGGGTGAC |  |
| CHIKn-For | CGTAATACGACTCACTATAGGACATCCTGCTCGGGTGAC | Negative strand |
| CHIKn-Rev | GGAATAATAGAGACTCCGAGAGG |  |
| Tagged strand-specific primers for cDNA synthesis (underlined is non-viral sequence followed by CHIKV nsP2 sequence) | | |
| PtagCHIKp | CGGGAAGGCGACTGGAGTGCCATGCTGCCTCGGGGTTAAATCCG | Positive strand |
| NtagCHIKn | GGCCGTCATGGTGGCGAATAAAGTCACCAAATGACCTTCG | Negative strand |
| Primers for qRT-PCR (underlines is non-viral sequence, others are CHIKV nsP2 sequences) | | |
| Ptag-For | CGGGAAGGCGACTGGAGTGCC | Positive strand |
| CHIKn-Rev | AGTCACCAAATGACCTTCGATAC |  |
| Ntag-For | GGCCGTCATGGTGGCGAATAA | Negative strand |
| CHIKp-Rev | ATGCTGCCTCGGGGTTAAATC |  |
