# Supplementary material for: Withaferin A inhibits Chikungunya virus nsP2 protease and shows antiviral activity in the cell culture and mouse model of virus infection
Source: PLoS Pathog. 2024 Dec 30;20(12):e1012816. doi: 10.1371/journal.ppat.1012816 (PMC11723598; doi:10.1371/journal.ppat.1012816)
Supplement: S3 Table — (DOCX) [file ppat.1012816.s009.docx]

**Table S3: The selection of residues at Site 1 and Site 2 for grid generation.**

| **Sites** | **Residues** |
| --- | --- |
| **Site 1** | C478, A511, Y512, S513, P514, E515, V516, Y543, Y544, N547, H548, W549, G555, K556, F558, Y666, N667, E669, L670, G671, P673, A674, M703, Q706, M707, G710, D711 and R714 |
| **Site 2** | Q504, A505, E508, K510, A511, Y512, G641, Y642, P656, L657, G658, V659, N667, L668, E669, I686, H687, T688, D700, H701, M703, K704, M707 and C698 |
