# Supplementary material for: Withaferin A inhibits Chikungunya virus nsP2 protease and shows antiviral activity in the cell culture and mouse model of virus infection
Source: PLoS Pathog. 2024 Dec 30;20(12):e1012816. doi: 10.1371/journal.ppat.1012816 (PMC11723598; doi:10.1371/journal.ppat.1012816)
Supplement: S4 Table — (DOCX) [file ppat.1012816.s010.docx]

**Table S4: SiteMap analysis highlights Site 1 as the potential WFA binding site.**

| **Title** | **SiteScore** | **size** | **Dscore** | **volume** | **exposure** | **enclosure** | **don/acc** |
| --- | --- | --- | --- | --- | --- | --- | --- |
| **Site 1** | **1.01** | **115** | **1.04** | **377.98** | **0.58** | **0.71** | **1.00** |
| Site 2 | 0.80 | 26 | 0.68 | 55.56 | 0.39 | 0.94 | 0.69 |
